# Rapid and convergent evolution of *Wolbachia* genomes in mosquito cell culture

**DOI:** 10.1371/journal.ppat.1014735

**Published:** 2026-10-23

**Authors:** Julien Martinez, Anne W. Wairimu, Steven P. Sinkins

**Affiliations:** MRC-University of Glasgow Centre for Virus Research, Glasgow, United Kingdom; Pennsylvania State University - Main Campus: The Pennsylvania State University - University Park Campus, UNITED STATES OF AMERICA

## Abstract

*Wolbachia* are widespread bacterial symbionts of arthropods that manipulate host reproduction and can confer fitness benefits, such as protection against viral pathogens. Their self-spreading ability coupled with their antiviral effect has been harnessed in health programmes to curb the transmission of dengue virus by *Aedes* mosquitoes. In this context, a better understanding of evolutionary dynamics over short timescales is critical to inform and improve the efficacy of *Wolbachia*-based disease control interventions. Moreover, experimental evolution of *Wolbachia* may provide a powerful approach for generating novel symbiont strains with improved properties for disease control. Here we generated new mosquito cell lines by introducing five *Wolbachia* strains from *Drosophila* into the symbiont-free *Aedes albopictus* Aa23 cell line. Following transinfection, long-read whole-genome sequencing revealed multiple mutations, including structural changes, some of them being associated with major shifts in bacterial density. Signs of convergent evolution, an excess of non-synonymous mutations and comparisons with previous studies, indicate that *Wolbachia* evolution in cell culture is predominantly driven by selection and occurs faster than in insect hosts. Altogether, our study demonstrates that cell culture is a valuable tool to investigate short-term symbiont evolution, explore the genetic basis of bacterial density regulation and generate higher-density symbiont strains suitable for disease control.

## Author summary

*Wolbachia* are bacteria that live inside many insects and can strongly influence their hosts, including reducing the ability of mosquitoes to spread viruses such as dengue. Because of this, *Wolbachia*-infected mosquitoes are now being released in several countries to help control mosquito-borne diseases. However, we still know very little about how these bacteria evolve or which genes control important traits, such as their abundance within the host. One major challenge is that *Wolbachia* cannot be grown outside insect cells, making genetic studies difficult.

Here, we developed an alternative way to study *Wolbachia* evolution by growing different strains in mosquito cell cultures and tracking how their genomes change over time. We found that *Wolbachia* evolved rapidly in cell culture, often acquiring similar genetic changes independently in different cell lines. Some of these changes were linked to large increases in bacterial abundance. Our results show that cell culture can be used to better understand how *Wolbachia* adapt and may eventually help generate improved bacterial strains for mosquito disease control.

## Introduction

*Wolbachia* is an intracellular, maternally-inherited bacterial symbiont found in a wide range of arthropods and some filarial nematodes [1]. Its maternal transmission is ensured by the symbiont’s ability to colonize the female germline and be passed on to the eggs. *Wolbachia* has evolved various kind of reproductive manipulations, such as cytoplasmic incompatibility, that promote the production of *Wolbachia*-carrying hosts in the population [2]. In addition to the germline, *Wolbachia* is often found in somatic tissues which can induce large effects on host fitness [3]. Notably, many *Wolbachia* strains inhibit the replication of viruses, a property that has been harnessed by health programmes around the world to control the spread of dengue virus by *Aedes* mosquitoes [4–12]. Higher *Wolbachia* densities within host cells generally provide stronger virus inhibition but can also induce substantial fitness costs by decreasing the host lifespan or fecundity [4,13,14].

While symbiont density regulation is central to the evolution of host-*Wolbachia* associations and the efficacy of *Wolbachia*-based disease control, the underlying genetic basis and mechanisms remain poorly understood. *Wolbachia* lives in the host cell cytoplasm within host-derived membranes and this obligate intracellular lifestyle has hampered the development of genetic tools to manipulate and study the symbiont’s genome [15]. As a result, *Wolbachia* research has largely relied on the characterization of natural genetic variation and comparative genomics to identify density-associated candidate genes and to guide the selection of symbiont strains for use in disease control [5,13,16–19]. This approach was successful in identifying Octomom, a 21 kb genomic region involved in density regulation in the pathogenic strain *w*MelPop, which overproliferates at high temperatures in *Drosophila melanogaster*, causing severe reductions in host longevity [20]. This overproliferating phenotype was linked to a higher Octomom copy number in *w*MelPop compared to the closely-related, lower-density strain *w*MelCS which carries a single copy of the region [13,21]. In *w*MelPop, Octomom copy number also varies between host generations, and increases with host age, with more copies being associated with a higher *w*MelPop density, pathogenicity and levels of virus inhibition [22–25]. This genomic instability is likely caused by the presence of repeat elements flanking the Octomom region, leading to the loss or the amplification of the region as a whole. Intriguingly, the complete loss of Octomom has similar effects to the amplification of the region by also increasing symbiont density, pathogenicity and virus inhibition compared to a single-copy variant, suggesting complex interactions between Octomom copy number and *Wolbachia* proliferation [14].

While Octomom provides a clear example of a density-regulating locus, symbiont density is likely governed by multiple biological pathways involving many genes that remain largely uncharacterized. Cases like Octomom in which *Wolbachia* strains maintained in the lab only differ at a small number of loci are rare, and comparative genomics approaches often yield long lists of candidate genes whose functional association with density remain hypothetical [16,17]. A complementary approach is to track genomic changes arising over short timescales. However, with the exception of specific genomic regions like Octomom in *w*MelPop and phage-associated reproductive parasitism genes in *Culex* mosquitoes that were shown to evolve rapidly [26–28], re-sequencing studies following the artificial transfer of the symbiont between host species or its introduction in the field for disease control have generally revealed a remarkable stability of *Wolbachia* genomes, even over periods spanning several years to more than a decade [16,17,29–31]. In contrast to insect studies, there is emerging evidence that *Wolbachia* may evolve rapidly when the symbiont is maintained in insect cell culture, potentially due to altered population dynamics and selective pressures in this environment. Woolfit et al. [21] observed a rapid burst of genomic changes during the passaging of *w*MelPop in mosquito cell lines, but detected no further mutations after subsequent transinfection into mosquitoes. These mutations included the complete loss of Octomom, the insertion of a transposable element and several non-synonymous mutations [21]. Similarly, in the closely-related *w*Mel strain, major genomic alterations were reported in a cell line derived from *D. melanogaster*, including the loss of Octomom and a ~ 3–4 fold amplification of a distinct 35 kb region [32].

Here we used an experimental evolution approach to investigate the evolution of several *Wolbachia* strains following their transfer from *Drosophila* into a common mosquito cell culture environment. Using whole-genome sequencing, we tracked the emergence and invasion dynamics of new mutations and observed their association with changes in symbiont density. Our results provide key insights into the evolution of *Wolbachia* genomes in the cell culture environment, new density-related candidate genes and demonstrate the utility of experimental evolution in cell culture for disease control applications.

## Results

### *Wolbachia* density fluctuations and complex population dynamics in cell culture

Five symbiont strains belonging to the arthropod-specific *Wolbachia* supergroup A were used to generate new *Wolbachia*-infected mosquito cell lines. These strains originated from various *Drosophila* species and were previously maintained in the same inbred line of *D. simulans* (STCP background) for six to more than 20 years depending on the strain (Table 1). *D. simulans* embryos were used as a source to infect *Wolbachia*-free *Ae. albopictus* Aa23 cells (see Methods). While all five *Wolbachia* strains successfully colonized mosquito cells (Fig 1A, S1A Fig), bacterial densities, estimated by qPCR as the ratio of bacterial-to-host target copy numbers, were highly variable across time. Although substantial short-term fluctuations were observed, overall *Wolbachia* density increased significantly throughout the experiment in all cell lines except the *w*Mel-infected line (S1A Table). In particular, *w*Tri-2-infected cells initially exhibited among the lowest symbiont densities for approximately 10 months, after which *w*Tri-2 became the highest density strain among the cell lines (Fig 1A).

**Table 1:** Origin of *Wolbachia* strains used for transinfection of Aa23 mosquito cells.

*Wolbachia* strain	Reference genome accession	Native host	Time spent in the *D. simulans* STCP line	References	Mean depth (Illumina)	Mean depth (Pacbio)
*w*Au	CP069055.1	*D. simulans*	~15 years	[33]	677x^b^	33x^f^
*w*Mel	CP046925.1	*D. melanogaster*	> 21 years	[34]	278x^b^, 135x^c^	11x^f^
*w*MelCS	CP046924.1	*D. melanogaster*	6 years	[4]	348x^b^	522x^f^
*w*Tri-2	CP195735.1^a^	*D. triauraria*	6 years	[4]	494x^d^, 206x^e^	6,434x^g^
*w*Tro	CP195734.1^a^	*D. tropicalis*	6 years	[4]	77x^b^	190x^f^

^a^*w*Tri-2 *and w*Tro reference genomes were sequenced and assembled from the Aa23 cell lines. ^b, c, d, e, f, g^ re-sequencing from the Aa23 cell lines after 17 (^b^), 27(^c^), 12(^d^), 22(^e^), 34(^f^) and 29(^g^) months post-transinfection.

**Fig 1:**
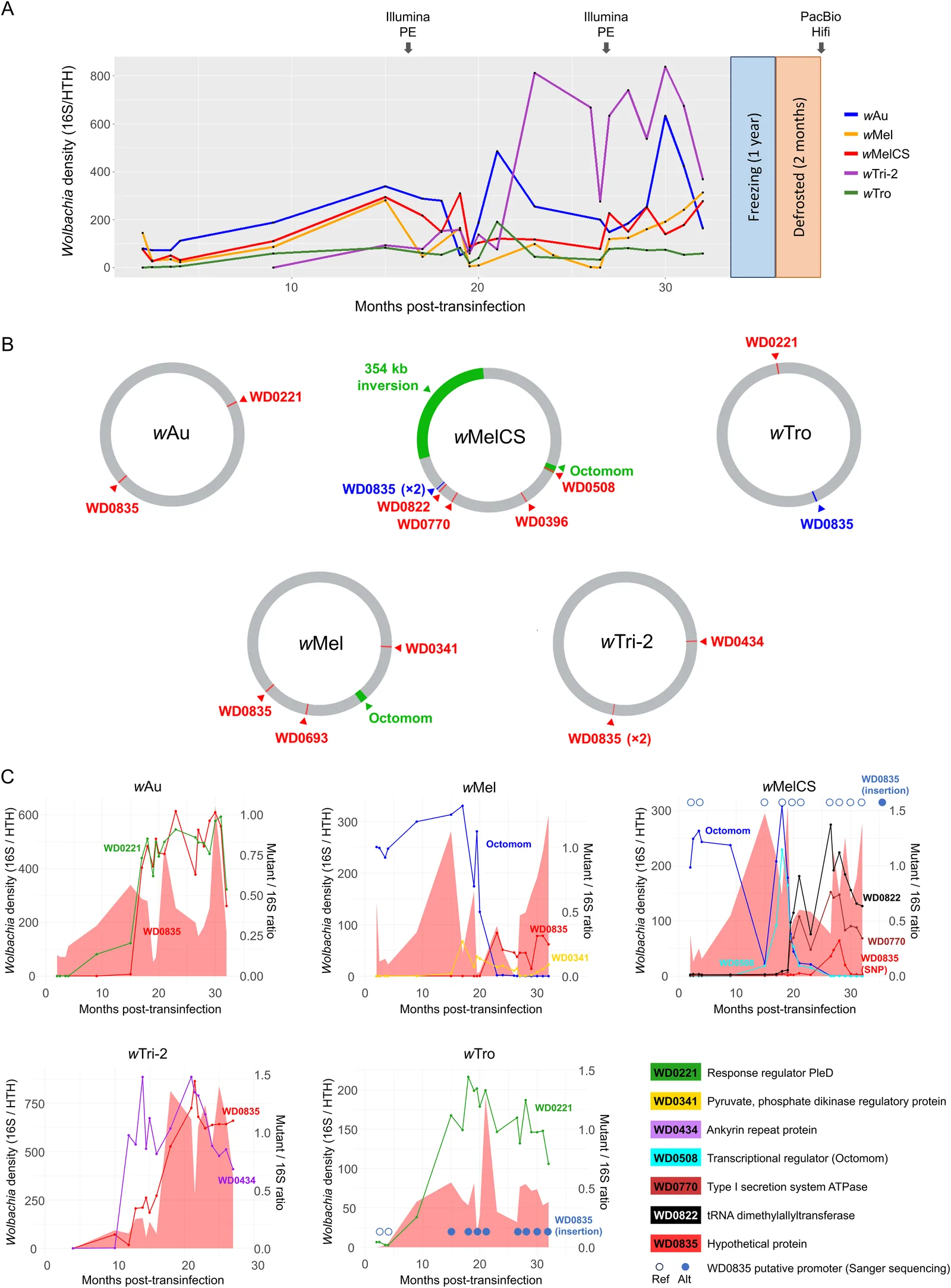
Changes in *Wolbachia* density and mutations in newly-created Aa23 cell lines. **(A)** Changes in *Wolbachia* density per cell line following transinfection. Lines indicate independent cell lines and each datapoint shows symbiont density in a given sample based on the mean of two technical qPCR replicates. The *w*Tri-2-infected cell line was generated five months after the other cell lines. Illumina sequencing was performed after 17 and 27 months in cell culture (12 and 22 months for *w*Tri-2). After 34 months (29 months for *w*Tri-2), all cell lines were frozen for one year and later defrosted for PacBio sequencing. **(B)** Summary of all mutations detected in each *Wolbachia* genome by comparing endpoint PacBio HiFi and intermediate-timepoint Illumina reads with the corresponding reference genomes from *Drosophila* hosts. SNPs and short indels are coloured according to their effect (red: non-synonymous; blue: putative regulatory region) and structural rearrangements shown in green. **(C)** Variation in *Wolbachia* density (red area; left axis) and estimated frequency of mutant alleles relative to the 16S rRNA control gene (right axis) measured by qPCR and averaged over two technical replicates. Genes affected by mutations are displayed in different colours. Circles indicate the presence of an ancestral (Ref) or mutant (Alt) allele for the insertion occurring in the AT-rich upstream region of gene WD0835, as determined by Sanger sequencing, since the qPCR approach failed.

Part of these fluctuations in *Wolbachia* density may be attributable to complex population dynamics or variation in other factors in the cell culture environment. For example, *Wolbachia* density was influenced by the host cell number seeded upon each passage to new flasks, with more cells generally leading to higher symbiont densities (S2A Fig). Higher *Wolbachia* densities also tended to be associated with a reduced growth rate of host cells, indicating that high-density *Wolbachia* strains could be selectively disadvantaged if *Wolbachia* was purely transmitted vertically upon host cell division (S2B Fig). However, symbiont fitness may be partially decoupled from the host fitness if the symbiont can also transmit horizontally between cells. We tested this hypothesis by co-culturing cells carrying one of the Supergroup A strains with cells harbouring the Supergroup B *w*AlbB strain native of *Ae. albopictus*. After 3 months, co-cultured *Wolbachia* strains were found within the same host cells, showing that, in addition to vertical inheritance, horizontal transmission contributes to *Wolbachia* persistence and dynamics in this cell culture model (S1B Fig).

### *Wolbachia* genomes accumulate non-synonymous and convergent mutations in cell culture

Besides the effects of population dynamics or environmental fluctuations, some changes in *Wolbachia* density could have been caused by evolutionary changes in the symbiont or host genomes following transinfection. In order to identify mutations in the symbiont, we re-sequenced the genomes of the transinfected *Wolbachia* strains after 29–34 months in cell culture using Pacbio Hifi reads. Complete genome assemblies were generated and compared with the ancestral genomes previously sequenced from the native *Drosophila* host species (Table 1; S2 Table; Fig 1B). Illumina paired-end sequencing was also used to track mutations at intermediate timepoints (Table 1; S2 Table). Large structural differences were investigated through whole-genome alignment and visual inspection of sequencing depth variation along the genomes (S3 Fig). Single Nucleotide Polymorphisms (SNPs) and short insertions and deletions (indels) were identified using a frequency threshold of 0.2 to detect intermediate to high frequency mutations. Finally, the invasion dynamics of the mutations across time were monitored using SNP-specific quantitative PCR at multiple timepoints (see Methods), or by Sanger sequencing where the qPCR approach failed (two mutations located in an AT-rich region upstream of gene WD0835 in *w*MelCS and *w*Tro). Using this range of approaches, we detected a total of 19 mutations across the five *Wolbachia* strains, with *w*MelCS showing the most changes.

The identified mutations included SNPs, short indels, large deletions, as well as a 354 kb chromosomal inversion in *w*MelCS (Fig 1B). Two SNPs in *w*Mel and *w*MelCS occurred within transposable elements (WD0693 and WD0396 genes), which are highly abundant in *Wolbachia* genomes and were not investigated further. For all other mutations, targeted Sanger sequencing failed to detect them in the *D. simulans* donor lines which were maintained in parallel throughout the experiment, suggesting that the mutations were either acquired or increased in frequency specifically in the cell-culture populations (S4 Fig). Most mutations were located within coding regions and induced non-synonymous changes, suggesting they may have phenotypic consequences. Many mutations spread from low to high frequencies, while others were transient or persisted at low to intermediate frequencies by the end of the experiment (Fig 1C).

Importantly, different *Wolbachia* genomes independently acquired mutations in the same genes or genomic regions. Such convergent mutations were observed in the WD0221 gene, a *PleD*-like response regulator, in the two closely-related strains *w*Au and *w*Tro (Fig 1B). Similarly, the Octomom region was lost both in *w*Mel and *w*MelCS. Finally, WD0835, which encodes a hypothetical protein, accumulated mutations in all the symbiont strains with SNPs and short insertions occurring within the coding region and in a putative AT-rich regulatory region directly upstream of the gene (see detailed analysis below). This pattern of convergent evolution combined with the excess of non-synonymous mutations strongly suggest that, at least some of the genomic changes were driven by selection rather than genetic drift.

### Repeated TE-mediated structural changes

All structural changes we identified occurred in the two closely-related strains *w*Mel and *w*MelCS and involved regions flanked by short transposable elements (TEs). The density-regulating Octomom region, which comprises eight genes flanked by group II intron reverse-transcriptase elements on each side, was lost as a whole unit in both strains, leaving behind a single copy of the TE (Fig 2A-C). This is reminiscent of the same mutations observed in previous studies when *w*MelPop was transferred to mosquito cell culture [21] and in *w*Mel following the generation of the *Drosophila* JW18 cell line [32,35]. In insects, the loss of Octomom was also identified in *w*MelCS during a genetic screening following the use of a mutagenic agent, which caused an increase in symbiont density [14]. In our *w*Mel and *w*MelCS cell lines, variation in average Octomom copy number was not significantly associated with changes in symbiont density, whether the effect of time was included or not in the model (S1A Table). Interestingly, during a strong rebound in Octomom copy number, in a phase of relatively high density, a mutation disrupting the Octomom gene WD0508 in *w*MelCS rose to high frequency before being eliminated together with the entire region (Fig 1C). While we observed no significant association between Octomom copy number and density in this experiment, environmental variation, complex population dynamics in cell culture and evolution of the host cell genome could have affected our ability to detect such a relationship. Moreover, endpoint PCR showed that polymorphism in Octomom copy number persisted for extended periods of time, indicating that our cell lines contained mixtures of genotypes carrying one or more copies of Octomom, or lacking the region entirely (S5 Fig). Finally, changes in density could have been affected by mutations in other genes that were segregating in the *w*Mel and *w*MelCS-infected cell lines (Fig 1C).

**Fig 2:**
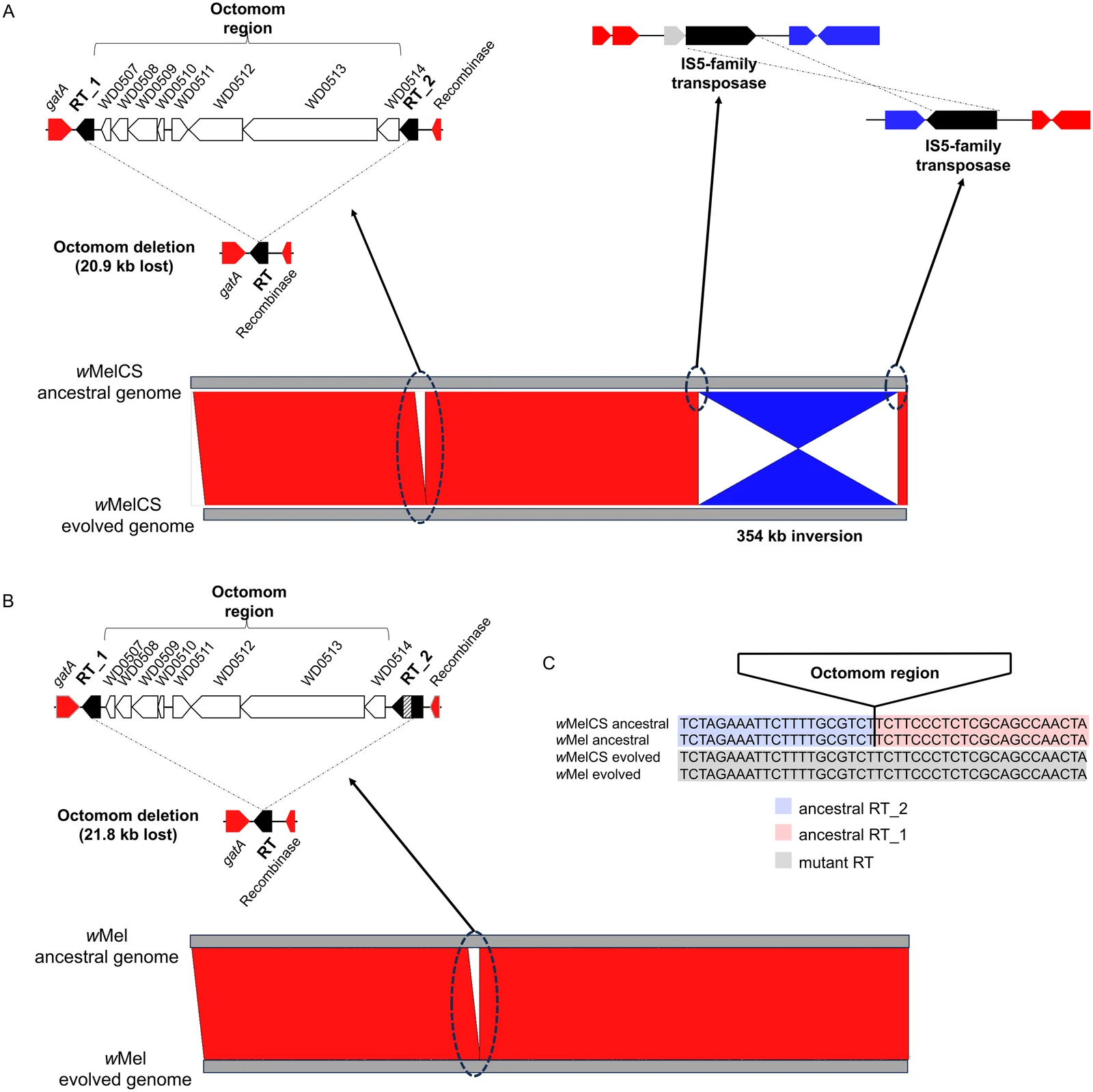
TE-mediated structural changes in *Wolbachia* genomes. **(A-B)** Whole-genome alignment of *w*MelCS (A) and *w*Mel (B) ancestral reference (CP046924.1, CP046925.1) and evolved (Pacbio; CP195736.1, CP195737.1) genome assemblies. The coloured areas between genomes indicate blocks of synteny based on blastn comparisons. Gene boxplots represent gene content at the sites of structural rearrangement. Black genes represent transposable elements (RT: group II intron reverse-transcriptase) and the dashed box in *w*Mel Octomom region indicates a transposase insertion disrupting the reverse-transcriptase. **(C)** Nucleotide alignment of reverse transcriptase genes flanking the Octomom region and at the deletion site. Note that the exact deletion site cannot be inferred since RT copies are identical in sequence.

To decouple these potential confounding factors from the effect of losing Octomom, we generated six additional *Wolbachia*-infected cell lines from flies for *w*MelCS, as well as its sister strain *w*MelPop in which Octomom is unstable, with copy number varying above one in *Drosophila* hosts [22]. To reduce stochastic effects, replicate cell lines were split in parallel at regular intervals (every ten days) for about 400 days. In this experiment, we observed evidence of both convergent evolution, with the same changes occurring independently in different *Wolbachia* genomes, and parallel evolution, where the same changes occurred in replicate cell lines derived from the same ancestral strain. In most replicates, Octomom copy number began to decline after around 200 days, concomitant with a significant increase in *Wolbachia* density (S6A Fig, S1B Table). When analysed separately, both time and Octomom copy number were significantly associated with density, with Octomom copies and density being negatively correlated (S6A Fig, S1B Table). However, when both predictors were included in the same model, Octomom copy number was no longer significantly associated with density. Because Octomom copy number was itself strongly correlated with time (*w*MelCS: *r* = -0.65, *p* < 0.0001; *w*MelPop: *r* = -0.73, *p* < 0.0001), the independent contributions of these two factors to changes in density could not be disentangled.

Nevertheless, although rebounds in the Octomom copy number were observed in some replicates, the overall decrease indicates that losing Octomom is selectively advantageous in cell culture. While Octomom copy numbers converged towards zero in most replicates, endpoint PCR showed that the region generally persisted at low frequency, as observed previously (S6B Fig). Interestingly, the drop in Octomom copy number was preceded by a transient increase above one copy in most replicates, suggesting that the presence of multiple homologous copies may provide additional opportunities for recombination, increasing the probability of generating deletion variants. Alternatively, the transient increases and subsequent declines may reflect changes in the relative frequencies of pre-existing copy-number genotypes, however, our data do not allow us to distinguish between these two hypotheses. Finally, the relative synchrony in the loss of Octomom across replicates suggests that some unknown environmental factor fluctuating over time may be driving the selective advantage of deleted genotypes. None of the other mutations identified in the original *w*MelCS cell line (Fig 1B) could be detected in the replicate *w*MelCS and *w*MelPop cell lines.

In addition to Octomom deletion, the large chromosomal inversion identified in *w*MelCS likely involved a TE-mediated structural rearrangement, as the region is flanked by two identical and pseudogenized IS5-family transposase-encoding elements (Fig 2A). As observed for Octomom, the ancestral and inversion variants coexisted for an extensive period (S5 Fig) and the inversion was detected independently in multiple *w*MelCS and *w*MelPop replicate cell lines (S6B Fig). Since flanking repeats were too large for designing specific qPCR primers, we were unable to track the temporal frequency dynamics of this inversion or directly assess its potential effect on symbiont density.

Neither Octomom deletion nor the chromosomal inversion could be detected in *D. simulans* or *D. melanogaster* hosts carrying *w*MelCS or *w*MelPop (S7 Fig). This suggests that TE-mediated recombination rates may be higher in cell culture or that such structural variants are strongly counter-selected in insect hosts.

### Shift in symbiont density associated with the evolution of two putative bacterial effectors

Besides the Octomom region, very few *Wolbachia* genes are known to affect symbiont density. Among the strains analysed here, *w*Tri-2 underwent a drastic and sustained increase in density during its evolution in Aa23 cells (Fig 1A). The only genomic changes detected in the *w*Tri-2 genome were two amino acid substitutions in gene WD0835 and a 5 bp insertion restoring the open reading frame of gene WD0434 which is truncated in the ancestral genome (Fig 1B and 1C). The invasion dynamics of these mutations suggest a causal link with the large increase in *w*Tri-2 density (Fig 1C). The WD0434 mutation rose to high frequency approximately five months before the sustained density increase, whereas the subsequent spread of the WD0835 mutant allele closely coincided with the phenotypic shift. These temporal patterns are consistent with several hypotheses. Since mutations in WD0835 in the other *Wolbachia* strains did not produce comparable density changes (Fig 1C), it is possible that it is the interaction between the WD0434 and WD0835 mutations that is responsible for the phenotypic change in *w*Tri-2. Alternatively, WD0434 might be solely responsible for this change but its impact on the population lagged behind the mutation’s spread.

Using a prediction tool for secreted proteins (https://bastionx.erc.monash.edu/[36],) we found that both WD0835 and WD0434 encode putative bacterial effectors, likely secreted through the Type IV secretion system present in *Wolbachia* genomes (S3 Table). WD0434 is a particularly strong effector candidate since it encodes a protein carrying ankyrin repeat domains shown to interact with multiple host targets [37,38]. Ankyrin repeat proteins are widespread in eukaryotes as well as symbiotic bacteria and viruses and often mediate protein-protein interactions [39]. In addition to ankyrin repeats, domain prediction analyses revealed that WD0434 contains both cytoplasmic and transmembrane domains (InterProScan, Fig 3A). Thus, the protein might localize either to the bacterial or host membranes, possibly exposing its outer membrane ankyrin domain to the host cytosol.

**Fig 3:**
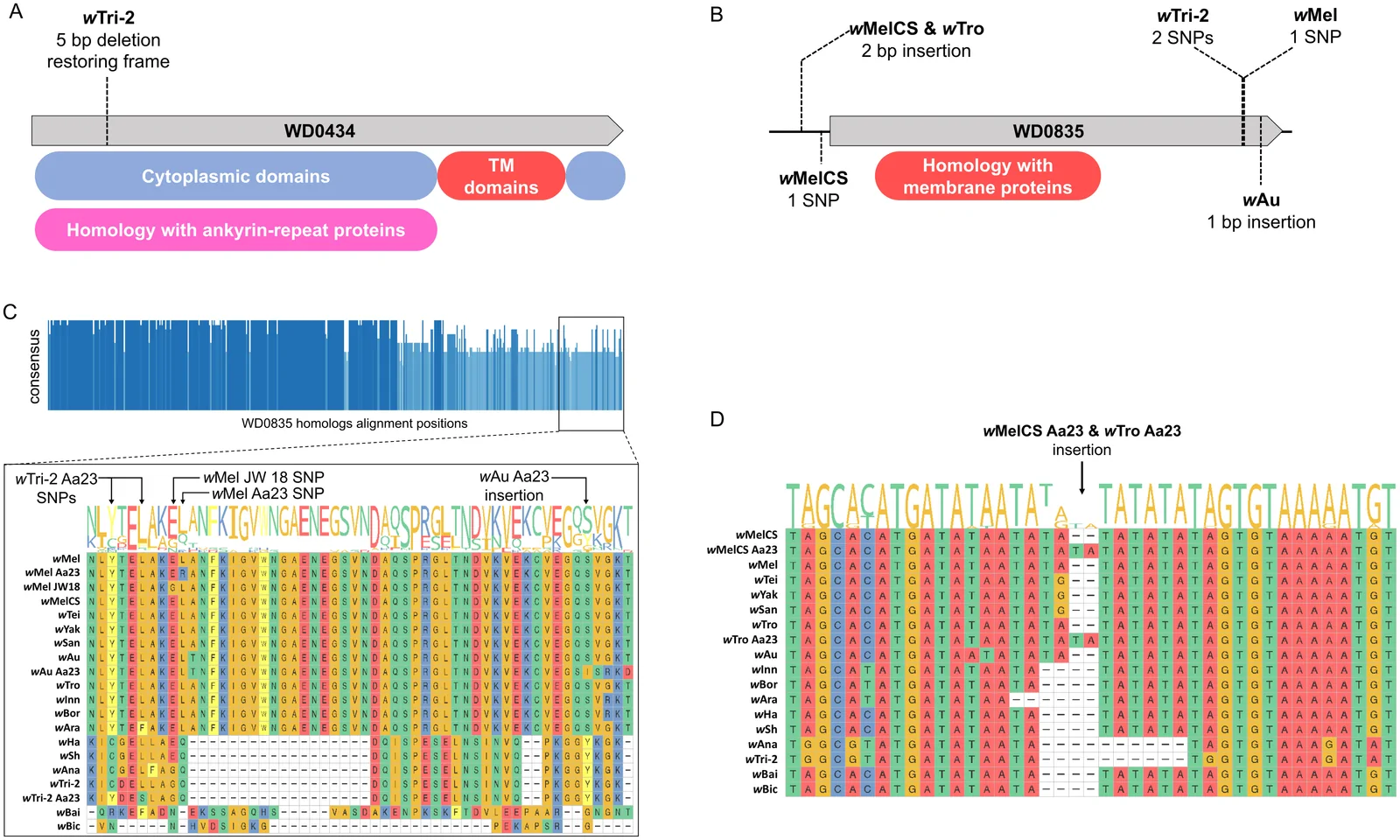
Comparative analysis of mutated genes in the *w*Tri-2 high-density mutant. **(A)** Mutations in WD0434 identified in the *w*Tri-2 cell line, predicted cytoplasmic and transmembrane domains (blue and red, Interproscan) and homologies to ankyrin-repeat containing proteins (pink, HHpred). **(B)** Mutations identified in WD0835 and its putative regulatory region across cell lines, and predicted homology to membrane proteins (red, HHpred). **(C)** Protein sequence alignment of WD0835 homologs visualized using the ggmsa visualization tool (http://yulab-smu.top/ggmsa/). Amino acids are coloured by chemistry in the zoom-in window (bottom); Blue bar chart: degree of sequence conservation across the protein alignment (top). **(D)** Nucleotide alignment of a putative AT-rich regulatory region located 81-120 bp upstream of WD0835.

WD0835 encodes a hypothetical protein which function is unknown, however, homology-based analyses revealed similarities to various eukaryotic proteins that interact with membranes and lipids, such as nuclear pore proteins, endoplasmic reticulum resident protein 29 or apolipoprotein E (HHpred probabilities: 91, 79 and 67% respectively, Fig 3B). These homologies suggest that WD0835 may interact with host membranes. Interestingly, WD0835 accumulated mutations in all our cell lines within two specific regions. First, in the C-terminal part of the protein with amino acid substitutions observed in both *w*Tri-2 and *w*Mel in the vicinity of each other, as well as a 1 bp insertion in the *w*Au homolog causing a frameshift adding 37 amino acids at the end of the protein. This pattern mirrors natural sequence variation observed among *Wolbachia* strains from insect hosts, in which the N-terminal region of WD0835 is highly conserved whereas the C-terminal region evolves more rapidly (Fig 3C). Notably, a publicly available *w*Mel genome sequenced from the *Drosophila* JW18 cell line (Genbank accession: CP186703.1 [35],) also carries a non-synonymous mutation located only a few base pairs from the mutation detected in our *w*Mel-infected Aa23 cell line (Fig 3C). Several mutations also occurred in an intergenic region directly upstream of WD0835 in both *w*MelCS and *w*Tro (Fig 3B). This includes an identical 2 bp insertion in both strains within an AT-rich sequence. This sequence varies substantially in length among *Wolbachia* strains isolated from insect hosts but is embedded within a more conserved region upstream of WD0835 (Fig 3D).

### Comparative analysis of density variation in an insect host

With the exception of the Octomom region, none of the candidate genes identified in our experimental evolution assay have previously been implicated in density regulation. Moreover, the specific gene(s) within the Octomom region responsible for density variation remain unclear. To investigate whether natural genetic variation in these genes is associated with differences in symbiont density, we compared the genomes of closely related *Wolbachia* strains previously shown to differ in density in *Drosophila* when measured in a controlled host genetic background (Fig 4; [4]).

**Fig 4:**
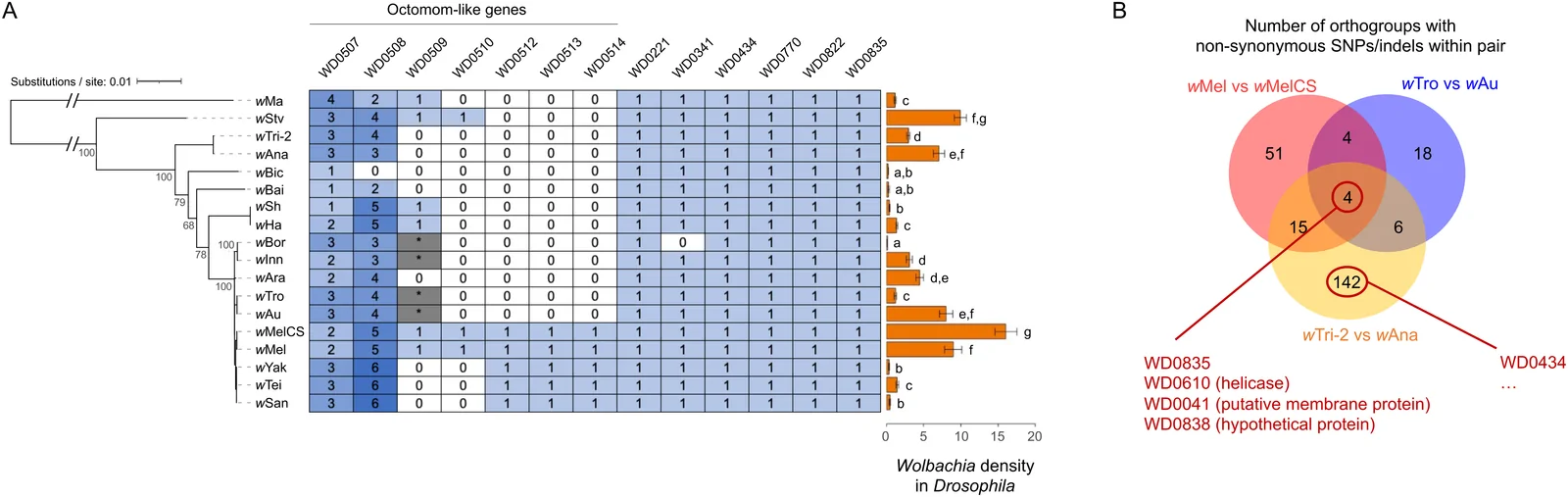
Comparative genomics of *Wolbachia* strains in *Drosophila.* **(A)** Left: maximum likelihood *Wolbachia* phylogeny based on 39 single-copy core genes with 1,000 bootstrap replicates (branch support). Center: presence (blue) and absence (white) of cell culture candidate genes across *Wolbachia* genomes. Numbers indicate numbers of homologs per genome (excluding putative pseudogenes) and grey indicates truncated versions of WD0509 homologs (pseudogenes). Right: *Wolbachia* density in whole flies. Error bars are standard errors and letters indicate statistically significant differences between strains based on a Tukey’s honest significance test on ln-transformed data (modified from [4]). **(B)** Venn diagram showing the number of orthogroups with non-synonymous variation within pairs of high/low-density sister strains. Overlaps indicate orthogroups shared between comparisons.

As reported previously, the complete Octomom region, comprising eight genes (WD0507–WD0514), is present as a single copy in *w*Mel and *w*MelCS, whereas an Octomom-like region lacking WD0509 and WD0510 homologs is found in *w*Yak, *w*Tei, and *w*San [16]. We also detected shorter Octomom variants in additional strains, most commonly lacking WD0510 and WD0512–WD0514 homologs (S8 Fig). Outside the canonical Octomom region, homologs of WD0507 and WD0508 are frequently located within prophage islands [40], and our comparative analysis revealed variable copy numbers of these genes across *Wolbachia* genomes (Figs 4A and S8). After excluding putative pseudogenes, neither the number of WD0507/WD0508 homologs per genome nor the presence of other Octomom-like genes was associated with symbiont density variation in *Drosophila* (S9A and S9B Fig). One exception was WD0510, whose presence was positively correlated with symbiont density. However, only three strains in our dataset harboured this gene, two of which are closely related (*w*Mel and *w*MelCS), limiting the strength of this association. Broader comparisons including additional strains carrying WD0510 will therefore be necessary to confirm this relationship. All other candidate genes identified in our experimental evolution assay (WD0221, WD0341, WD0434, WD0770, WD0822 and WD0835) were present as single copies in all strains, except for WD0341, which was absent in *w*Bor, and showed no association with symbiont density (Fig 4A).

In addition to gene content, we examined whether coding sequence variation was associated with differences in density. We selected three pairs of closely related *Wolbachia* strains exhibiting significant differences in density in *Drosophila*, as previously shown in [4]: *w*MelCS/*w*Mel, *w*Au/*w*Tro, and *w*Ana/*w*Tri-2. Most genes harbouring non-synonymous SNPs between high- and low-density sister strains were unique to a single pair of strains (Fig 4B; S4 Table).

Interestingly, four genes harboured non-synonymous SNPs in all three pairs of strains, one of which was WD0835 (Fig 4B). Within each pair, WD0835 SNPs were all localized to the C-terminal variable region of the protein (Fig 3C), with the exception of an additional N-terminal SNP distinguishing *w*Tri-2 and *w*Ana. These two strains also differed by two non-synonymous mutations in WD0434, one corresponding to the short deletion that restored the reading frame in the *w*Tri-2 homolog in our cell lines, and another SNP located near the end of the protein (S4 Table). The presence of SNPs and indels in both WD0434 and WD0835 between low- and high-density sister strains is consistent with a potential role for these genes in density regulation, as suggested by our cell culture evolution experiment. No other SNPs within pairs of *Wolbachia* strains were detected among our candidate genes identified in cell culture.

### Differences in evolutionary rate between insect and cell culture models

To place our results in a broader context, we compared the patterns of *Wolbachia* genome evolution observed here with those reported in previous studies examining *Wolbachia* evolution following host transfers, maintenance in insect cell culture, or field deployment for disease control (S10 Fig). To ensure consistency across studies, raw sequencing data were reanalysed using our variant detection pipeline (S5 Table). In most cases, no mutations were detected following *Wolbachia* transfer between insect host species or genetic backgrounds (Fig 5 and S10, S5 Table). The only exception was *w*Yak in which 4 coding and 3 non-coding mutations were reported between its native host *D. yakuba* and two independently transinfected lines of *D. simulans*. However, it is unclear whether the *w*Yak isolate sequenced from *D. yakuba* was the same that was transferred into *D. simulans.* In particular, the presence of the same 7 mutations in both replicate *D. simulans* lines would require identical changes to have arisen independently, which is unlikely.

**Fig 5:**
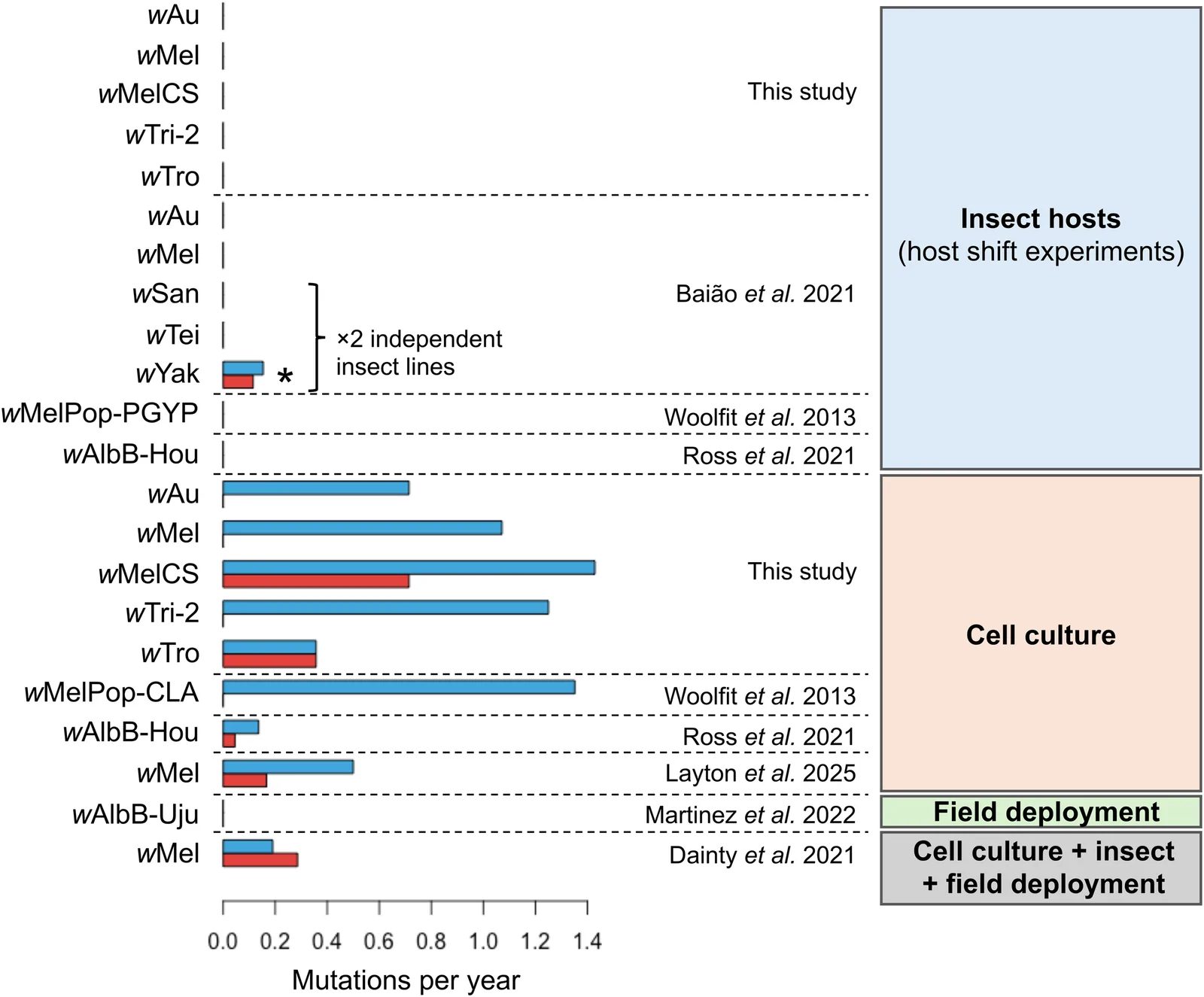
Variation in evolutionary rates between cell culture and insect studies. Blue bars: non-synonymous mutations. Red bars: synonymous mutations. *: the origin of the two re-sequenced *w*Yak lines is unclear and the mutations detected may have predated the generation of these lines in the lab. For consistency across studies, structural changes such as Octomom loss were excluded from this analysis, as long-read sequencing data was not available for all the re-sequenced *Wolbachia* strains.

In stark contrast to insect host transfers, multiple mutations were consistently detected during passaging of *Wolbachia* in insect cell lines, with the majority resulting in non-synonymous changes within coding regions. By normalizing mutation counts by the time spent in a new insect host or cell culture, we estimated that *Wolbachia* genomes accumulate on average 0.15 coding and 0.12 non-coding mutations per year in a new insect host, while in cell culture, they accumulate 6.8 coding mutations and only 1.28 non-coding mutations per year (Fig 5 and S10). This striking difference indicates a substantially accelerated and distinct mode of genome evolution in the cell culture environment.

Finally, we examined two studies that tracked *Wolbachia* evolution following field release of the strains *w*AlbB-Uju and *w*Mel into *Aedes aegypti* mosquito populations (Fig 5 and S10). In the case of *w*AlbB-Uju, no mutations were detected after two years in the field. In *w*Mel, five mutations (2 coding and 3 non-coding) were found across multiple mosquito populations, segregating at low to intermediate frequencies. However, it remains unclear whether these mutations occurred in the field or during the two years of passaging of *w*Mel in a mosquito cell line that preceded its field release [11].

## Discussion

Due to its obligate intracellular lifestyle, *Wolbachia* is not cultivable outside host cells which has greatly limited genetic studies. Insect cell culture has therefore become a widely used system to investigate *Wolbachia* biology, using both transinfected or naturally-infected cell lines [32,41–47]. However, to our knowledge, only one study has explicitly examined the temporal dynamics of *Wolbachia* genome evolution in cell culture [21], and this approach has not been exploited to identify candidate genes underlying variation in symbiont density. Here, we generated new *Wolbachia*-infected mosquito cell lines and identified mutations, providing new insights into the evolutionary dynamics of *Wolbachia* in cell culture. While we cannot exclude the possibility that these variants were present at low frequency in the *Drosophila* donor populations, the identified mutations were initially undetectable and generally rose in frequency only after prolonged passage in cell culture. Together with the repeated occurrence of similar changes in independent cell lines, this pattern is more consistent with selection contributing to their spread during adaptation to the cell culture environment.

### Gene functions and putative roles in density regulation

Overall, *Wolbachia* density increased in most of our cell lines during experimental evolution. Although changes in the symbiont genome may have contributed to this pattern, our experimental design does not allow us to establish direct causal relationships between individual mutations and changes in density. Other factors may have influenced symbiont density, including fluctuations in uncontrolled environmental conditions or changes in the relative abundance of infected and uninfected host cells. In addition, because host-cell populations evolved alongside *Wolbachia*, changes in the host genome may have occurred and interacted with the evolving symbiont. Our qPCR-based estimates also reflect the ratio of bacterial to host DNA rather than a direct measure of bacterial abundance per cell and could be affected by changes in representation of the host target, for example through shifts in host-cell ploidy. Functional validation will therefore be required to determine whether the mutations identified here directly affect *Wolbachia* density. Such validation could include side-by-side comparisons of ancestral and derived symbiont genotypes in a common, controlled host genetic background, ideally complemented by independent measurements of bacterial abundance at the cellular level. Nevertheless, the recurrent occurrence of mutations at several loci, together with their temporal association with density shifts, identifies a set of candidate genes whose potential roles in symbiont density regulation warrant further investigation.

The Octomom region was consistently lost in our cell lines, mirroring observations from previous cell culture studies [21,32]. Both the deletion and amplification of Octomom are known to increase *Wolbachia* density in *Drosophila* hosts. Consistent with these findings, the loss of Octomom in our cell lines tended to be associated with increases in *Wolbachia* density. However, the statistical support for this relationship weakened when time was included in the same models and also varied among experiments. Several factors may explain these results. First, Octomom copy numbers changed strongly over time, making it difficult to distinguish the effect of Octomom loss from that of other time-dependent processes. In experiments where the association was non-significant even without accounting for time, limited replication (single cell line and fewer timepoints, Fig 1) may also have reduced our statistical power. In addition, Octomom-related variation might not be fully captured by population-average copy number alone. Our cell lines comprised mixtures of deleted, single-copy and amplified Octomom genotypes that coexisted for extended periods, and the relationship between mean copy number and density may be non-linear in polymorphic cell lines, as both deleted and amplified variants are expected to reach higher densities than single-copy variants. Moreover, long-read sequencing was performed only at the end of the experiment and we cannot exclude the presence of low-frequency or more complex structural variants within Octomom at intermediate timepoints or in the fly donor populations that may have affected density. Finally, Octomom is unlikely to be the sole determinant of density. Previous work has shown that *Wolbachia* density regulation also depends on factors such as temperature and symbiont genetic background, independently of Octomom copy number [48]. Therefore, mutations in other density-regulating loci may have contributed to density changes and obscured the specific contribution of Octomom in some cell lines. In addition, the relative synchrony of changes among replicate *w*MelCS and *w*MelPop cell lines suggests that some shared environmental factors have influenced *Wolbachia* density, Octomom copy number or both (S6 Fig). Thus, while our results are consistent with Octomom loss contributing to increased *Wolbachia* density, they do not allow us to quantify its effect independently of other genetic and environmental changes occurring during prolonged cell culture.

Nevertheless, Octomom is known to affect density in fly hosts, however, the contribution of each of the eight genes within this region remains unclear [13,14,22–24]. Some of them encode proteins with putative roles in DNA replication, repair or transcription and could directly affect bacterial proliferation, while others harbour eukaryotic-like or toxin domains and may act as bacterial effectors [13,49,50]. In *w*MelCS, we observed the spread of a mutation introducing a premature stop codon in one of the Octomom genes, WD0508, suggesting that it has lost its function. The spread of this mutation coincided with a transient but substantial rebound in average Octomom copy number. Because the mutation occurred within an Octomom-bearing genotype, an increase in the frequency of that genotype would also increase the population-average Octomom copy number. Thus, the temporal association is compatible with selection favouring the WD0508-bearing genotype, although our data do not establish that the WD0508 mutation itself was responsible for its increase in frequency. The WD0508 protein harbours DNA-binding helix-turn-helix motifs homologous to transcription regulators and might regulate the expression of other *Wolbachia* genes. Alternatively, WD0508 could be a secreted effector involved in *Wolbachia*-host interactions since its transgenic expression in *Drosophila* increases *Wolbachia* density [51].

Beyond Octomom, WD0835 emerged as a strong candidate regulator of symbiont density. Mutations in WD0835 accumulated in all mosquito cell lines and, in the case of *w*Tri-2, were associated with a pronounced increase in *Wolbachia* density. Although another mutation in the ankyrin repeat-encoding gene WD0434 segregated in the same cell line and could have contributed to this shift in density, the timing of the phenotypic change was more strongly correlated with the spread of the WD0835 mutant allele. Recent work has shown that WD0434 can bind specific host proteins and cause severe toxic phenotypes upon heterologous expression, however its effect on symbiont density is yet to be demonstrated [38]. WD0835 encodes a hypothetical protein previously identified as a putative bacterial effector based on its phylogenetic distribution being restricted to the *Wolbachia* genus [37]. We found that its conserved N-terminal region harbours structural homologies to various membrane and ER-associated proteins, pointing to a potential role in *Wolbachia*’s interactions with host membranes. In addition, the recurrent changes in the AT-rich sequence upstream of WD0835 in our cell lines, together with similar variation among naturally occurring *Wolbachia* strains, raise the possibility that this sequence is part of a rapidly evolving regulatory region controlling WD0835 expression. Further work comparing WD0835 expression between symbiont strains or mapping transcription start sites using approaches such as Cappable-Seq [52] will be needed to test this hypothesis. Interestingly, WD0835 expression pattern throughout the host development is tightly correlated with that of a neighbouring gene, WD0830, with high levels of expression in *Drosophila* larvae and pupae [37,53]. WD0830 encodes a secreted effector named WalE1 that interacts with the host actin cytoskeleton and increases symbiont density when heterologously expressed [54,55]. Therefore, the WD0835 protein might act in concert with WalE1 to regulate symbiont proliferation through interactions with the host. As independent support for the role of WD0835 in density regulation, our comparative analysis of *Wolbachia* strains from a previous study in *Drosophila* also identified this gene as a top candidate to explain density variation between closely-related strains [4]. However, the persistence of *w*Tro at low density compared to other strains, despite the emergence of a mutation in the upstream region of WD0835 demonstrates that changes at this locus are not sufficient, on their own, to produce a high-density phenotype. Their effects may depend on the precise mutation and the symbiont and host genomic backgrounds. Nevertheless, the recurrent targeting of WD0835 suggests that this locus may contribute to adaptation to cell culture independently of any direct effect on symbiont density.

Our study also identified additional candidate genes that deserves further characterization to test their potential role in density regulation. For instance, WD0221 mutated in both *w*Au and *w*Tro, two strains that showed increasing densities over time (Fig 1C). WD0221 encodes the response regulator of a two-component PleC/PleD signalling pathway that regulate many processes in bacteria. While its role in *Wolbachia* remains uncharacterized [56], homologous systems in other intracellular bacteria are thought to play an important role in host cell infection [57,58].

### Role of mobile elements in structural variation

Previous studies have highlighted the important role of TEs in *Wolbachia* evolution and our experiment largely confirmed this [14,22,26,27,59–61]. For instance, TEs within phage-associated genomic islands have been proposed to promote the amplification and diversification of reproductive parasitism genes in *Culex* mosquitoes [27]. Both the Octomom region and the large chromosomal inversion we identified are flanked by identical TEs and have repeatedly evolved in our cell lines. Comparative and phylogenetic studies suggest that TE-associated evolution is partly attributable to their transpositional activity and horizontal transfer between *Wolbachia* genomes [59,62–64]. However, a large proportion of *Wolbachia* TEs are also predicted to be nonfunctional [62], including the two pseudogenized IS5-family elements flanking the chromosomal inversion identified here. Their identical sequences could nevertheless provide substrates for homologous recombination. Thus, in addition to active transposition, recombination between TE sequences may be an important mechanism generating structural variation in *Wolbachia* genomes.

The repeated spread of the chromosomal inversion indicates that it may be advantageous in cell culture, although with the current data we cannot establish whether this impacted symbiont density. Nevertheless, this is reminiscent of the global replacement of *w*MelCS by the lower-density strain *w*Mel reported in natural populations of fruit flies [61]. The two strains, while genetically very similar, can be distinguished by a large inversion flanked by IS elements. While this inversion occurs at a different genomic position than the 354-kb inversion identified here, the two cases highlight a common capacity of TEs to mediate large-scale genome restructuring.

### Selection versus genetic drift in the cell culture environment

Consistent with previous observations [21], nearly all mutations we identified induced non-synonymous changes in *Wolbachia* genes suggesting they are likely to have functional consequences. The few exceptions occurred in a putative regulatory region which may affect gene expression levels and therefore the associated phenotype. Many mutations reached and maintained high frequencies, and some genes mutated independently across multiple cell lines. Although genetic drift cannot be excluded, these patterns suggest that selection played a major role in shaping *Wolbachia* genome evolution in cell culture. The absence of synonymous mutations may reflect a combination of low mutation rate (estimated to be ~ 100-fold lower than the mitochondrial mutation rate in *Drosophila* [65]) and limited recombination relative to the speed of selective sweeps when beneficial mutations occur. Because *Wolbachia* reproduces clonally, the fixation of a mutation is expected to purge standing variation across the genome, erasing linked genomic diversity at other loci in the symbiont population.

Despite strong evidence for selection, in some cases we detected the persistence of polymorphism over extended periods of time. For example, Octomom, which was repeatedly lost, often persisted at very low frequency across replicates. This may be due to fluctuating selective pressures in the cell culture environment. In particular, the Aa23 cell line is thought to be heterogenous, being composed of different cell types with distinct morphologies [42], and competition for resources likely varies over time due to repeated cycles of host cell growth and stationary phases during passaging. Finally, variation in host cell density may alter the balance between vertical and horizontal transmission of *Wolbachia*. Under such conditions, polymorphism may be maintained if the relative fitness of symbiont haplotypes depends on host cell type, intracellular competition, or opportunities for horizontal transfer.

Evolutionary theory predicts that within hosts, overproliferating symbionts should have a competitive advantage, even at the expense of the host’s fitness, a phenomenon called the “tragedy of the commons” [66,67]. For vertically-transmitted symbionts, this may also increase the probability of transmission to the next generation. However, at the between-host level, overproliferating mutants should be counter-selected if they induce strong fitness costs on their host, as observed for *Wolbachia* in insects [13,14,22,68]. In Aa23 cell lines, high *Wolbachia* densities were associated with reduced cell growth, suggesting a similar trade-off due to conflicting selective pressures within and between host cells. However, we also found that *Wolbachia* is transmitted horizontally between cells, consistent with previous findings in both cell culture and insect studies [69–71]. If horizontal transmission is sufficiently high, host and symbiont fitness may be largely decoupled, allowing costly overproliferating variants to persist or even be favoured in cell culture.

### Evolution in cell culture versus the insect model

All the mutations we characterized in our cell lines were undetectable in the *Wolbachia* genomes sequenced from their natural *Drosophila* host species and from the *D. simulans* lines used for transinfection. Although low-frequency ancestral variants cannot be excluded, this suggests that these *Wolbachia* strains underwent little genomic change over 6–21 years spent in a novel insect host background, yet accumulated multiple mutations over much shorter timescales in cell culture. In line with this, our meta-analysis also showed a consistently higher rate of genomic change in cell culture than in studies conducted in insect hosts.

These observations suggest that the evolutionary dynamics of *Wolbachia*, and particularly the relative roles of selection and genetic drift, differ markedly between the two environments. In insects, maternal transmission imposes severe genetic bottlenecks at each host generation, reducing effective population sizes and increasing the influence of genetic drift. In contrast, in cell culture, *Wolbachia* can be inherited during host cell division while also spreading horizontally between cells. This mixed mode of transmission is expected to lead to higher effective population sizes, thereby reducing the relative influence of drift and increasing the efficacy with which beneficial variants can rise in frequency.

Another key difference is likely to lie in the traits and genomic regions exposed to selection. In our cell lines, we detected no mutation within prophage regions or associated genes involved in reproductive parasitism, despite evidence that these loci can evolve rapidly in insect hosts and natural populations [26,27,72–74]. This may reflect relaxation of selection on reproductive parasitism in cell culture, where host sexual reproduction is absent. Conversely, genes affecting intracellular proliferation of the symbiont may be under strong selection in cell culture, if horizontal transmission of *Wolbachia* between cells is highly advantageous.

Thus, the contrast between cell culture and insect hosts is unlikely to simply reflect a difference in the overall rate of evolution. Rather, these environments appear to differ both in the efficiency and the targets of selection. Cell culture may therefore lead to evolutionary trajectories that differ qualitatively from those observed in natural host-symbiont associations.

### Experimental evolution in cell culture to optimize symbionts for disease control

The deployment of antiviral *Wolbachia* strains for the control of mosquito-borne viruses has primarily focused on the release of two moderate-density strains *w*AlbB and *w*Mel and has resulted in drastic reductions in dengue cases at release sites [6,7]. However, despite the proven efficacy of the technique, there have been instances where *Wolbachia* failed to establish or persist in the field [75,76], likely due to strain-dependent phenotypes that were not fully characterised before release [77]. For example, the *w*Mel strain is more sensitive to heat stress than alternative strains [78], and temperature fluctuations or heatwaves can eliminate the bacteria within a population. Without a method for targeted modification of the genome, the optimisation of *Wolbachia*-based disease control has relied on identifying naturally occurring strains which show potential as vector control tools. The rapid evolution of *Wolbachia* strains in cell lines reported in this study provides a potential alternative pipeline for generating novel mutant strains with desirable characteristics, such as higher intracellular densities or improved heat tolerance. The emergence of high-density variants such as *w*Tri-2 in cell culture illustrates the potential utility of this approach for expanding the repertoire of candidate strains available for vector control.

### Conclusions

Whether *Wolbachia* genomes evolve faster in cell culture due to larger population sizes or because selective pressures are intrinsically different in this environment remains an open question. Further work is also needed to validate the causal links between the mutations we characterized and changes in symbiont density. Nevertheless, our study demonstrates that the experimental evolution in cell culture is a promising approach for functional genetics of *Wolbachia* in the absence of genetic transformation tools. Our findings also have important implications for *Wolbachia*-based disease control applications, whose long-term efficacy depends on the stability of the symbiont’s antiviral phenotype [79,80]. Finally, the cell culture model could be a powerful tool to study *Wolbachia*-host coevolutionary dynamics. While our study focused on changes in the symbiont genome, evolution of the host may also have occurred and could contribute to the observed density dynamics, which is an important avenue for future investigation.

## Methods

### *Wolbachia* strains, fly husbandry and cell transinfection protocol

The *Wolbachia* strains used for transinfection were sourced from the *D. simulans* STCP background (Table 1), except for *w*MelPop which was sourced from the *D. melanogaster* DrosDel *w*^1118^ isogenic background [13]. All flies were maintained at 18°C on a cornmeal diet under a 12-hour light/dark cycle. Twenty-four hours before transinfection, a 96-well plate was seeded with *Wolbachia*-free Aa23 cells (~1.6 × 10^5^ cells per well) in Schneider’s media (10% Fetal Bovine Serum, 1% PenStrep) and cells were left to attach to the bottom of the well overnight. On the day of transinfection and for each *Wolbachia* strain, *Wolbachia*-infected adult flies were allowed to lay eggs in BugDorm cages (W17.5 x D17.5 x H17.5 cm) with a Petri dish containing agar (3% agar, 1% sucrose, water) and a spot of yeast paste in the centre to stimulate egg-laying. After 1 hour, 500–1,000 fly embryos were collected from the Petri dish with a metal spatula, transferred to a 50 ml Falcon tube and rinsed in 50 ml of sterile water three times. Fly embryos were allowed to sink and the embryo pellet transferred to a 1.5 ml Eppendorf tube. Embryos were then surface-sterilized in 1 ml of 50% commercial bleach solution for 2 min and rinsed in 1 ml of 70% ethanol for 5 min twice and finally 1 ml of sterile water. Embryos were then resuspended in 1 ml of Schneider’s media, mechanically crushed and homogenized with a sterile pestle to release intracellular *Wolbachia*. The embryo homogenate was centrifuged at 2,500 g for 10 min to remove eggshells and cell debris. 100 µl of the supernatant was overlaid onto Aa23 cells. Once the mosquito cells formed a confluent monolayer covering the base of the well, mosquito cells were serially passaged from the 96-well plate, to 48-, 24- and 12-well plates. Cells were later maintained in 25 cm^2^ flasks in Schneider’s media at 28°C and transferred to new flasks every 7–10 days by diluting 1 mL of cell suspension into 4 mL of fresh media (1 in 5 dilution). The cell lines were frozen in liquid nitrogen 34 months post-transinfection (29 months in the case of *w*Tri-2). After one year, the cell lines were defrosted and maintained in flasks as described above for about 2 months before Pacbio sequencing (see below). Cultures were inspected routinely by microscopy for evidence of bacterial or fungal contamination. In addition, Markadoros (https://github.com/sanger-tol/markadoros) was used to assemble and taxonomically classify small subunit (SSU) ribosomal RNA markers to assess the presence of contaminants in the Pacbio sequencing data. Assembled SSU were also compared against the NCBI nt database, and no SSU markers other than *Wolbachia* 16S and mosquito ribosomal sequences were detected (S7 Table).

### Quantification of *Wolbachia* density

For each sample, DNA was extracted from ~1.6 × 10^6^ Aa23 cells resuspended in 200 μL of STE buffer followed by a 30 min incubation at 65˚C with 2 μL of Proteinase K (20 mg/mL) and a final incubation for 10 min at 95˚C. For DNA extraction from flies, the same was done by homogenizing a single adult female in STE buffer with a sterile pestle. The extracted DNA was then diluted 1/5 in water and samples were centrifuged at 2,000 g for 2 min before endpoint PCR or qPCR.

*Wolbachia* density relative to host DNA was measured by qPCR using primers amplifying the single copy *Wolbachia* 16S rRNA gene and the mosquito Homothorax gene (HTH) as a control ([81], S6 Table). For each gene, 2 μL of DNA template and the Fast SYBR Green Master Mix (Applied Biosystems) were used in 10 μL reactions: 5 μL of master mix, 2 μL of water, 0.5 μL of each 5 μM primer and the following cycles: 95˚C for 20 s, 40 cycles of 95˚C for 1 s and 60˚C for 20 s, followed by a melt-curve analysis. For each sample and each gene, two qPCR reactions were done. *Wolbachia* density was then estimated by averaging the Cq values of the two technical replicates per gene and the following formula: *2*^*(mean HTH Cq - mean 16S Cq)*^.

Changes in *Wolbachia* density over time were analysed separately for each strain using linear regression in R [82]. Since density measurements showed evidence of non-normality and heteroscedasticity, Box–Cox analyses were used to identify suitable transformations with the boxcox function in the R package MASS. A square-root transformation (λ ≈ 0.5) was supported across *Wolbachia* strains and was therefore applied. For the experiment containing several replicate cell lines of the same *Wolbachia* strain (*w*MelCS and *w*MelPop), temporal changes in density were analysed using linear mixed-effects models, with symbiont density as the response variable, timepoint as a continuous fixed effect, and replicate cell line identity as a random effect. The significance of fixed effects was assessed using *t*-tests using the lmer function as implemented in the R package lmerTest [83]. Where appropriate, the effect of Octomom copy number on density was tested, both alone and in combination with the timepoint effect (S1 Table).

### *In situ* detection of *Wolbachia*

Cells were plated out overnight in a poly-L-lysine coated 24-well glass-bottom plate (Corning, New York, USA) at a density of 5 × 10^5^ cells per well. Schneider’s media was then removed and cells were fixed with 200 µL of ice cold 10% formalin for 30 minutes at room temperature. Cells were washed twice in PBST (PBS, 0.1% Tween 20) then incubated overnight at 37°C in a hybridisation buffer containing 50% formamide, 25% 20X SSC, 0.2% dextran sulphate, 2.5% herring sperm DNA, 1% tRNA, 1% Denhardt’s solution, 0.015% DTT and 100 ng/ml of each *Wolbachia* 16S rRNA probe. To detect all transinfected symbiont strains, the following *Wolbachia* probes were combined: W2 5’-CTTCTGTGAGTACCGTCATTATC-(Cyanine3)-3’, W3 5’- AACCGACCCTATCCCTTCGAATA-(Cyanine3)-3’ [9]. In order to distinguish the transinfected Supergroup A strains (*w*Au and *w*Mel) from *w*AlbB in the co-culture experiment, we combined a Supergroup A-specific probe (5’-ACCTGTGTGAAACCCGGACGAAC-(Alexa fluor 488)-3’) with a *w*AlbB-specific probe (5’-TAGGCTTGCGCACCTTGCAGC-(Cyanine3)-3’). Following this, samples were washed twice in a solution with 5% 20X SSC, 0.015% DTT and twice in 2.5% 20X SSC, 0.015% DTT for 20 minutes per wash at 55°C. Cells were then stained for 20 minutes with Hoechst 33342 (ThermoFisher, MA, USA) at a concentration of 1 µg/mL in PBS. Finally, cells were washed with PBS and left in PBS for visualisation to prevent drying out. All samples were visualised on a Zeiss LSM 880 confocal microscope (Zeiss, Oberkochen, Germany).

### *Wolbachia* purification and DNA preparation for sequencing

To ensure enough DNA material for sequencing, a population of Aa23 cells was derived from each of the *Wolbachia*-infected cell lines and maintained in Schneider’s media in two 225 cm^2^ flasks per symbiont strain. In order to increase the *Wolbachia*-to-host DNA ratio, cells were passaged twice in 225 cm^2^ flasks every 10 days using a 1 in 2 dilution, instead of 1 in 5, as higher cell density was found to increase *Wolbachia* density (S2 Fig). Cells from the two flasks were then harvested and concentrated by centrifugation at 2,500 g for 2 min. The cell pellets were pooled, resuspended in 2 mL of Schneider’s media and homogenized with 1mm beads in 2 mL screw cap tubes in a tissue lyzer. The homogenate was then centrifuged at 2,000 g for 2 min to remove cellular debris. For Pacbio sequencing, DNA was extracted directly from the supernatant with the Gentra Puregene tissue kit (Qiagen) and the DNA pellet resuspended in 100 µL of nuclease-free water. For Illumina sequencing, intact host cells and cellular debris were removed before DNA extraction by passing the supernatant through 5 and 2.7 µm pore size filters. The filtrate was then centrifuged at 18,500 g for 15 min to pellet the bacteria. The supernatant was discarded and the bacterial pellet resuspended in 1 mL of Schneider’s media and the previous centrifugation step repeated once. The final resuspended bacterial pellet was then incubated with 20 µL of DNase I at 37°C for 30 min to digest remaining host DNA. Following digestion, the sample was centrifuged at 18,500 g and the supernatant discarded. DNase I was inactivated by incubating the sample at 75°C degrees for 10 min and DNA was then extracted as explained above.

### Whole-genome sequencing, assembly, variant and sequencing depth analysis

Pacbio libraries were prepared using the Revio SMRTbell prep kit (PacBio) and sequenced on a Revio SMRT 25M ZMW cell in HiFi mode. DNA libraries for Illumina sequencing were prepared using the Kapa LTP Library Preparation Kit (KAPA Biosystems, Roche7961880001) and sequenced on the Illumina MiSeq platform with the MiSeq Reagent Kit v3 to generate 2 × 150 bp reads. Raw Illumina reads were demultiplexed using bcl2fastq and adapters were trimmed with Trimmomatic v0.38.0 [84]. Host reads were filtered out by mapping the Illumina and Pacbio Hifi reads against the *Ae. albopictus* reference assembly (Genbank accession: GCA_006496715.1) using BWA-MEM v0.7.17 [85] and Minimap2 v2.23 [86] respectively. Unmapped Pacbio reads were assembled using Hifiasm v0.19.8 [87].

For variant analysis, host-decontaminated Pacbio and Illumina reads were mapped to their respective *Wolbachia* reference genome and to the Pacbio-assembled genomes from the Aa23 cell lines with Minimap2 and BWA-MEM. Publicly available Illumina reads that were used to assemble the ancestral reference *Wolbachia* genomes were mapped in the same way. In the case of *w*Mel, we ran comparisons to the most recent reference genome [14], since [31] showed that the older version [88] contains multiple errors which led to the detection of false-positive variants (one SNP and seven short indels) in several studies investigating the evolution of this strain [16,29,31]. Reads mapped onto the symbiont genome were filtered with bamtools v2.5.2 [89] to remove duplicates and retain properly paired reads with a minimum mapping quality of 20 (MAPQ), except for reads with a MAPQ score of 0 in order to keep reads mapping to repetitive regions. The SNP analysis was then conducted using the Snippy pipeline v4.6.0 (Seeman, Torsten. Snippy: fast bacterial variant calling from NGS reads; 2020 https://github.com/tseemann/snippy). SNPs were called with a threshold of 10 reads minimum coverage, 0.2 minimum proportion, > 30 Phred quality scores for variant evidence (i.e., 99.9% accuracy)(S2 Table). Since some of our Pacbio read datasets had low sequencing depth (Table 1), we also ran Snippy in “contig” mode, which generated artificial 250 bp single-end reads at 100x coverage from the Pacbio-assembled sequences in order to call variants. Detected variants were visually inspected by loading the BAM alignment files into the Artemis genome viewer [90]. We discarded five SNPs in *w*Au that were detected both using reads from our *w*Au-infected Aa23 cell line and those used to assemble the *w*Au reference genome (S2 Table). These were all located in intergenic repeat regions, some showing signs of within-sample polymorphism, indicating they are most likely sequencing errors in the *w*Au reference genome. The sequencing depth per base was also calculated for each BAM file with the samtools depth command [91] and visualised with the R software [83]. Finally, structural variants were analyzed by performing blastn whole-genome alignments and visualizing genome synteny in the Artemis comparison tool.

### Timecourse analysis of mutations

SNPs and short indels were first validated by Sanger sequencing using PCR products generated with primers listed in S6 Table. The timecourse analysis of the identified mutations was then conducted using qPCR and SNP-specific primers. To this end, primer pairs were designed with one of the primers having the last or the penultimate base corresponding to the mutated allele on the SNP (S6 Table). To improve specificity, an artificial mismatch was added at the 3’ end of some primers, creating a double-mismatch with the ancestral allele [92]. PCR efficiencies were assessed with a dilution series (S11A Fig) and the specificity of the amplification was validated by ensuring that no or negligible amplification was observed for samples carrying the ancestral allele (S11B Fig). The copy number of the target SNP relative to 16S was calculated from the average of two technical replicates per sample as follows: *2*^*(mean 16S Cq – mean SNP Cq)*^. Relative SNP copy number was then used as a proxy for the frequency of the SNP in the symbiont population. Copy number variation for the Octomom region (WD0513) was calculated in the same way. Since WD0508 had several paralogs, we designed PCR and qPCR primers within highly divergent regions of the gene, allowing us to specifically validate the mutation in WD0508 by Sanger sequencing and to accurately track its frequency in the cell lines.

In order to test for within-sample polymorphism in Octomom copy number, endpoint PCR with primers amplifying the WD0513 gene and primers flanking the Octomom region was conducted to detect the Octomom-carrying and deleted variants respectively (S6 Table). To detect the presence of the large chromosomal inversion in *w*MelCS and *w*MelPop, endpoint PCR primers were designed on one side of the inversion, in the flanking region, with one primer pair amplifying the inversion variant and one pair the non-inverted ancestral allele (S6 Table). The *wsp* gene was used as a control for *Wolbachia* presence. Each set of primers was used in separate reaction with 2 μL of DNA template and the 2X DreamTaq Green PCR Master Mix (ThermoFisher) in a 21 μL reaction: 9.4 μL of master mix, 9.4 μL of water, 0.1 μL of each 20 μM primer. The following PCR cycle was used: 95˚C for 3 min, 35 cycles of 30s denaturation at 95˚C, 30s for primer annealing at 56 ˚C, 3 min extension at 72˚C and a 15 min final extension step at 72˚C.

### Comparative genomics of *Wolbachia* from *Drosophila*

All genome sequences were reannotated with Prokka v1.14.6 using the default parameters [91] and clusters of orthologous proteins across the eighteen genomes were inferred with Orthofinder v2.5.5 [93]. In order to refine our analysis for pairs of low- and high-density symbiont sister strains, we used Roary v.3.13.0 [94] with a 95% identity threshold to split homologous groups containing paralogs into groups of true orthologs. The Roary output was manually curated to fix issues with wrongly annotated pseudogenes in the accessory genome by visualizing the genome annotations in Artemis [90]. Pseudogenized repeat elements (transposases and reverse transcriptases) were excluded from the manual curation due to their large number and the difficulty to infer orthology between genomes. SNPs and small indels between genomes of a pair of sister strains were called with Snippy as explained above. The *Wolbachia* phylogeny was inferred from a set of 39 single-copy core genes as determined by Roary using RAxML-NG v1.0.2 [95].

## Supporting information

S1 Fig Fluorescent *In situ* Hybridization imaging of *Wolbachia*-infected Aa23 cell lines. (A) Singly-infected Aa23 cell lines conducted 2 months post-transinfection. (B) Comparison of singly- and coinfected Aa23 cell lines after 3 months of passaging using 16S rRNA probe A (*w*Au/*w*Mel-specific, green) and probe B (*w*AlbB-specific, red).
(TIF)

S2 Fig Relationship between *Wolbachia* density and host cell growth rate. Six cell lines originating from the same flasks were derived from either *w*Au- (left) or *w*Tri-2-infected (right) mosquito cells. Three replicate lines per *Wolbachia* strain were first passaged at low cell density (~8 × 10^5^ cells: blue, black, purple) and the three other lines at high cell density (~1.6 × 10^6^ cells: orange, red, brown) for four passages before the start of the experiment. Several flasks per replicate cell line were then seeded with 1.6 × 10^6^ cells and *Wolbachia* density (A) and mosquito cell concentration (B) were measured every two days (each datapoint corresponds to a different flask).
(TIF)

S3 Fig Sequencing depth of *Wolbachia* genomes in Aa23 cells. (A) Paired-end Illumina reads. (B) Pacbio Hifi reads. Sequencing depth was averaged over 500 bp windows.
(TIF)

S4 Fig Validation of detected mutations in *Wolbachia* genomes. (A) Sanger sequencing chromatograms of SNPs and small indels identified in transinfected *Wolbachia* strains in *D. simulans* and their Aa23 cell line counterpart at the end of the experimental evolution assay. (B) Alignment of Sanger-sequenced PCR product with wMelCS 5’ end chromosomal inversion breakpoint.
(TIF)

S5 Fig Endpoint PCR analysis of structural rearrangements in *w*Mel and *w*MelCS. Gel electrophoresis of PCR products for detecting Octomom and chromosomal inversion variants in samples collected at different timepoints following transinfection into Aa23 cells. The *wsp* gene was used as a positive control for *Wolbachia* presence. The *w*MelCS- and *w*Mel-infected *D. simulans* STCP line were used as positive controls (+). NC: H_2_O negative control. L: DNA ladder.
(TIF)

S6 Fig Repeated loss of the Octomom region and chromosomal inversion in replicate cell lines. (A) Relative Octomom copy number across time estimated by the qPCR amplification of the WD0513 homolog (left panels) in *w*MelCS- (top) and *w*MelPop-infected (bottom) cell lines. Middle panels show the corresponding *Wolbachia* densities across time. Right panels show the relationship between Octomom copy number and *Wolbachia* density, with the trend lines, *R*^*2*^ and *p*-values indicating the fixed effect of Octomom copy number estimated from a mixed effect model excluding the effect of time. (B) Gel electrophoresis of PCR products for detecting Octomom variants and chromosomal inversions in samples collected 410 days post-transinfection (290 days for *w*MelCS replicate 4 which was lost after that). The *wsp* gene was used as a positive control for *Wolbachia* presence. L: DNA ladder. NC: H_2_O negative control.
(TIF)

S7 Fig Absence of the chromosomal inversion in *Drosophila* lines. (TIF)

S8 Fig Octomom region and homologs among *Wolbachia* strains in *Drosophila.* Colours indicate homology between Octomom-like genes, transposable elements (black) or other genes (grey).
(TIF)

S9 Fig Tests of association between Octomom-like genes and *Wolbachia* density in *Drosophila.* Datapoints represent the mean symbiont density in whole flies as measured in (Martinez et al. 2014).
(TIF)

S10 Fig *Wolbachia* transinfection history and genome re-sequencing. (TIF)

S11 Fig Efficiencies and specificity of qPCR primers used. (A) 1 in 5 dilution series. Each datapoint is the mean of two technical replicates. Red line: regression model used to estimate qPCR efficiencies of each primer pair. (B) qPCR validation of SNP-specific primer specificity. Single adult female flies were used for *Wolbachia*-infected *D. simulans* donor lines. Samples collected after 27 months (22 months for *w*Tri-2) post-transinfection are shown for *Wolbachia*-infected Aa23 cells, except for the WD0508 SNP in *w*MelCS for which samples collected between 15–20 months post-transinfection are shown.
(TIF)

S1 File Uncropped electrophoresis gel images corresponding to Supplementary Figs S5–S7. Full uncropped ethidium bromide-stained agarose gels are shown for the PCR assays used to characterize *Wolbachia* genotypes in the *w*Mel, *w*MelCS and *w*MelPop-infected cell lines and fly hosts (PCR primers listed in S6 Table). Sample identities are indicated above each gel with numbers indicating timepoints (S5 Fig), replicate cell lines (S6 Fig) or individual fly hosts (S7 Fig). L: DNA ladder; + and −: positive and negative controls respectively.
(PDF)

S1 Table Statistical analyses of *Wolbachia* density changes with time and Octomom copy number. (XLSX)

S2 Table SNP analysis of *Wolbachia* strains in Aa23 cell lines. (XLSX)

S3 Table Predictions of secreted proteins using BastionX. (XLSX)

S4 Table SNP analysis of *Wolbachia* strains from *Drosophila.* (XLSX)

S5 Table SNP analysis of previous insect and cell culture studies. (XLSX)

S6 Table PCR and qPCR primers. (XLSX)

S7 Table BLASTN analysis of small subunit (SSU) ribosomal RNA markers in Pacbio data. (XLSX)
