# Supplementary material for: Rapid and convergent evolution of Wolbachia genomes in mosquito cell culture
Source: PLoS Pathog. 2026 Oct 23;22(10):e1014735. doi: 10.1371/journal.ppat.1014735 (PMC13719037; doi:10.1371/journal.ppat.1014735)

S5 Fig. Electrophoresis gel; migration time: 35 min;
dye: Ethidium bromide; strain: wMel; target: wsp

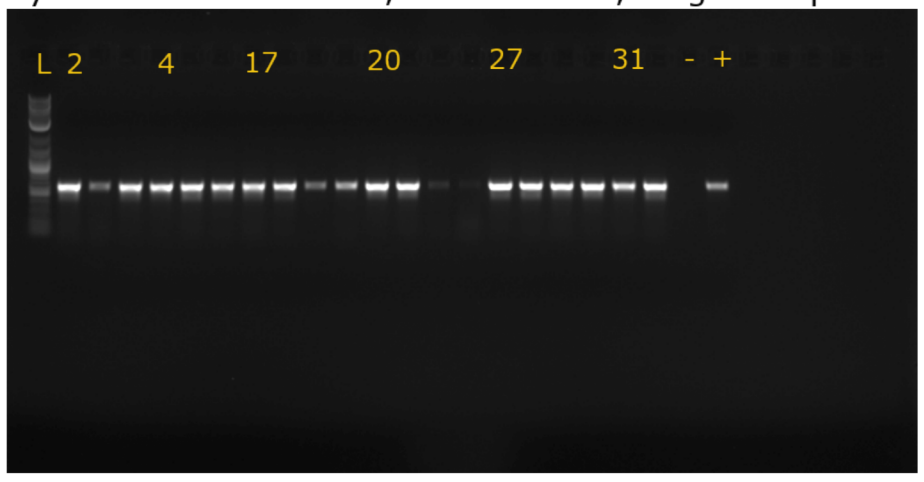

S5 Fig. Electrophorsesis gel; migration time: 35 min;
dye: Ethidium bromide; strain: wMelCS; target: wsp

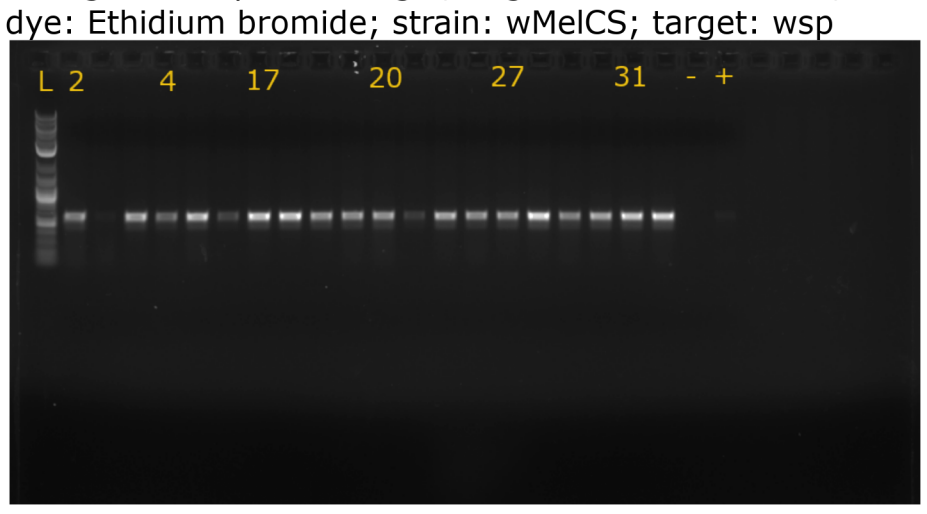

S5 Fig. Electrophoresis gel; migration time: 35 min;
dye: Ethidium bromide;strain: wMel; target: Octomom deletion

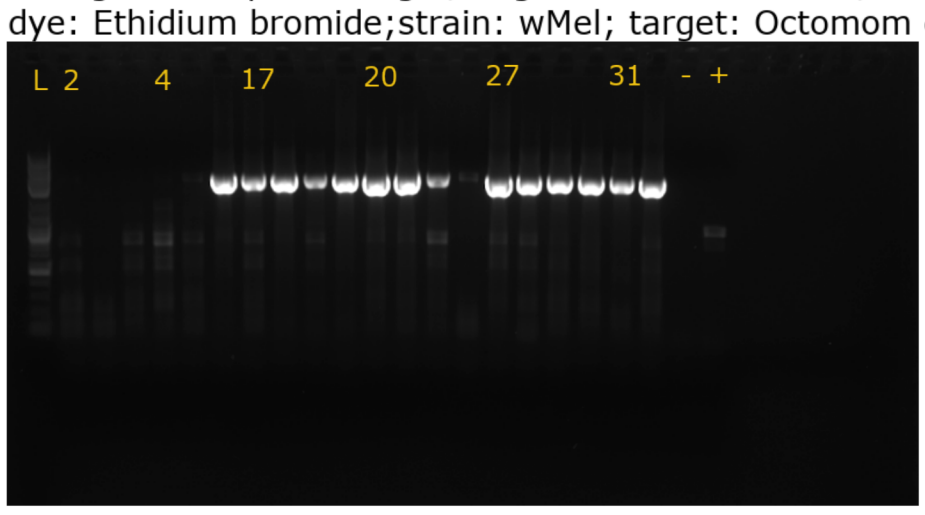

S5 Fig. Electrophoresis gel; migration time: 35 min;
dye: Ethidium bromide;strain: wMelCS; target: Octomom deletion

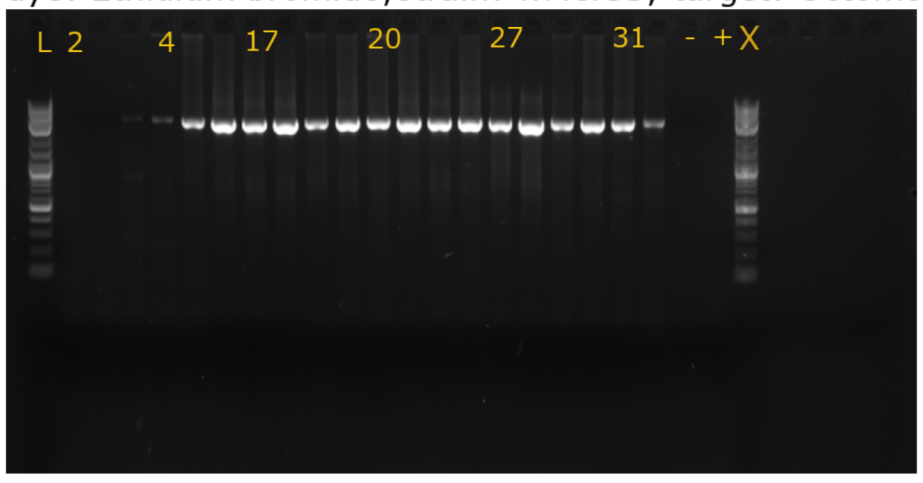

S5 Fig. Electrophoresis gel; migration time: 35 min;
dye: Ethidium bromide;strain: wMel; target: WD0513

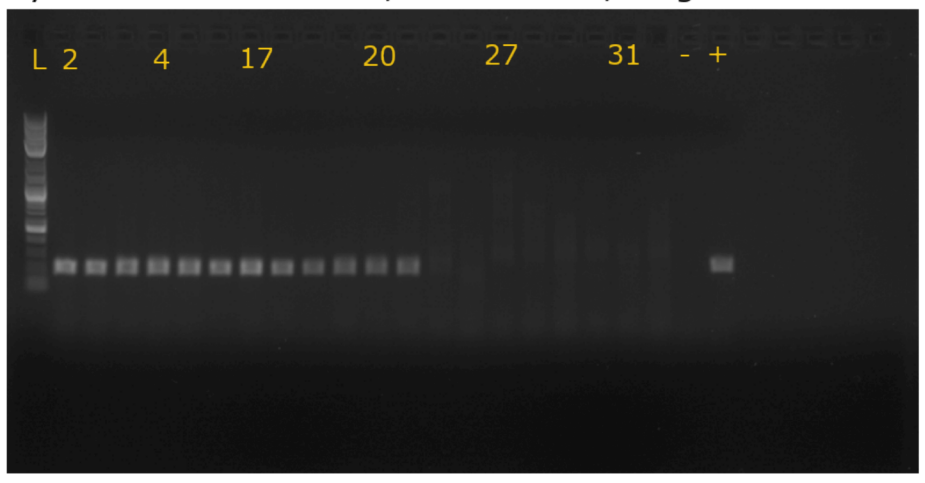

S5 Fig. Electrophoresis gel; migration time: 35 min;
dye: Ethidium bromide;strain: wMelCS; target: WD0513

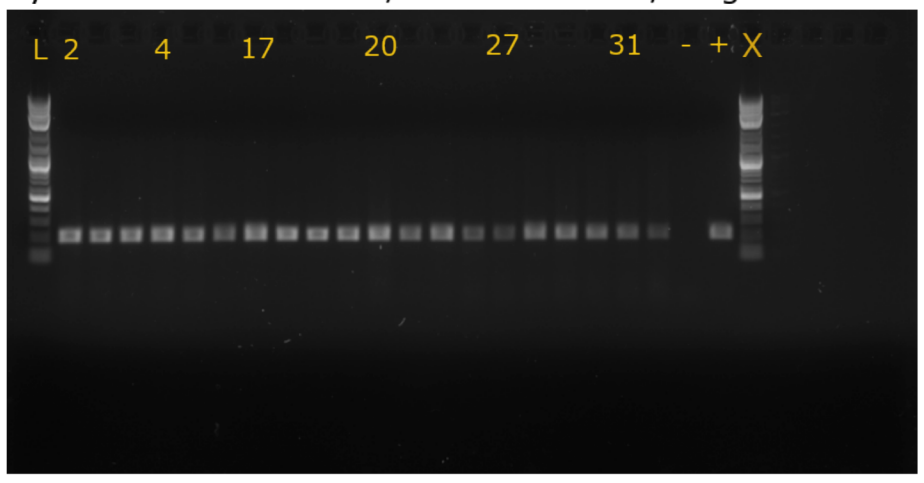

S5 Fig. Electrophoresis gel; migration time: 35 min;
dye: Ethidium bromide;strain: wMelCS; target: inversion mutant

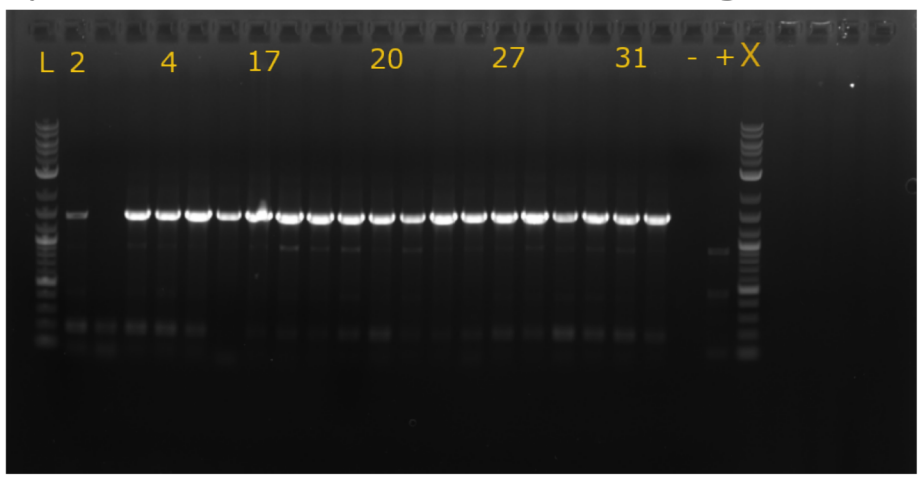

S5 Fig. Electrophoresis gel; migration time: 35 min;
dye: Ethidium bromide;strain: wMelCS; target: inversion reference

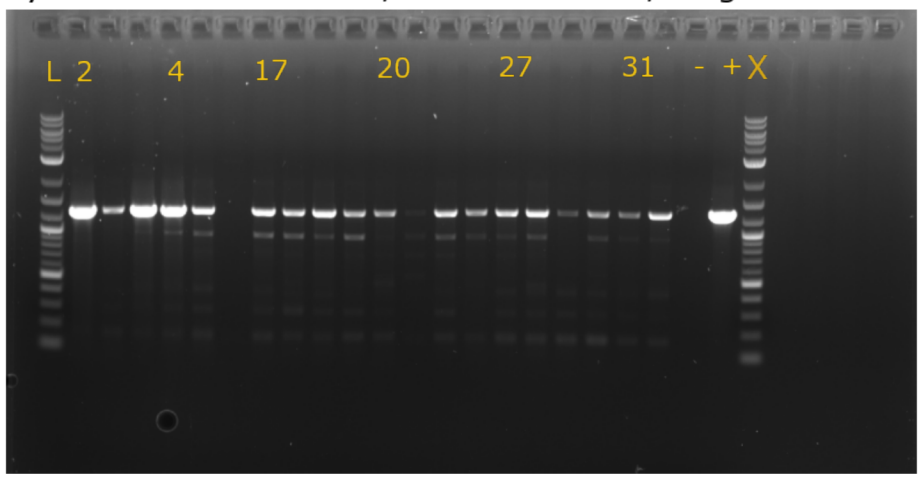

S6 Fig. Electrophoresis gel; migration time: 35 min;
dye: Ethidium bromide;strain: wMelCS & wMelPop; target: wsp

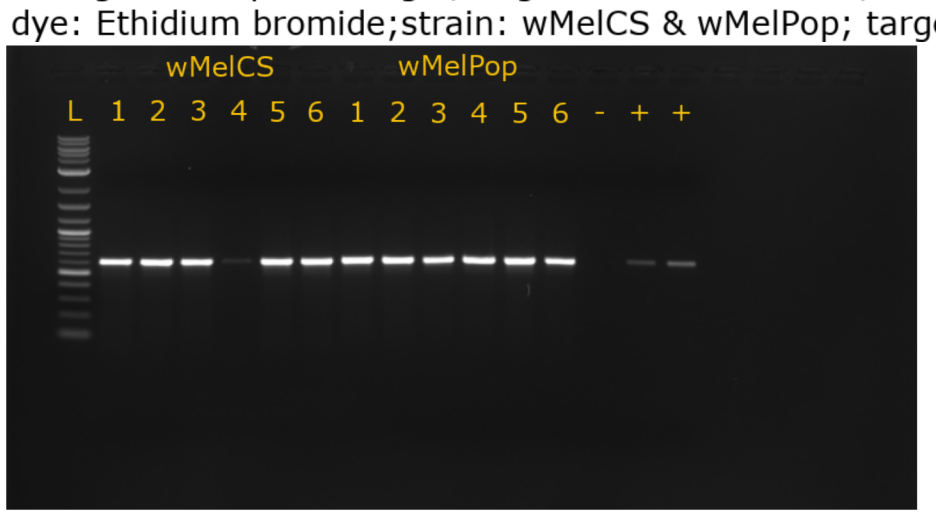

S6 Fig. Electrophoresis gel; migration time: 35 min;
dye: Ethidium bromide;strain: wMelCS & wMelPop; target: Octomom deletion

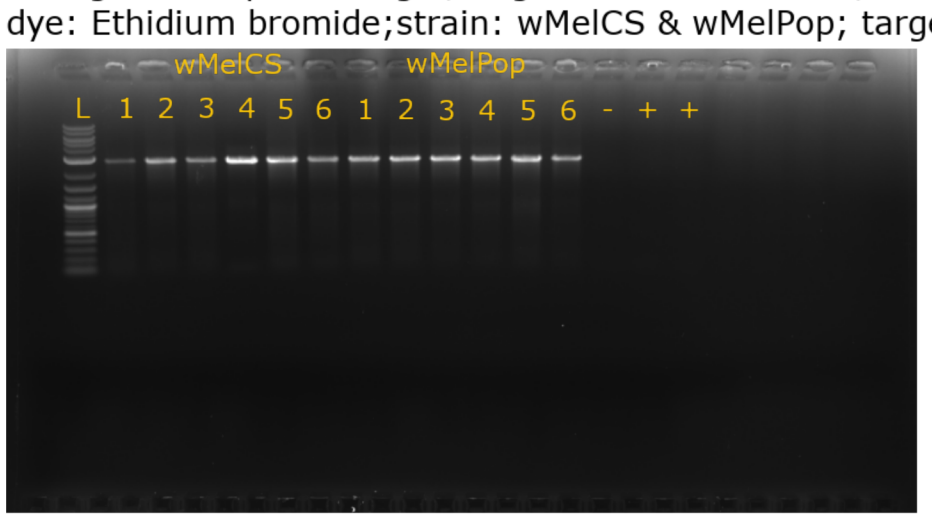

S6 Fig. Electrophoresis gel; migration time: 35 min;
dye: Ethidium bromide;strain: wMelCS & wMelPop; target: WD0513

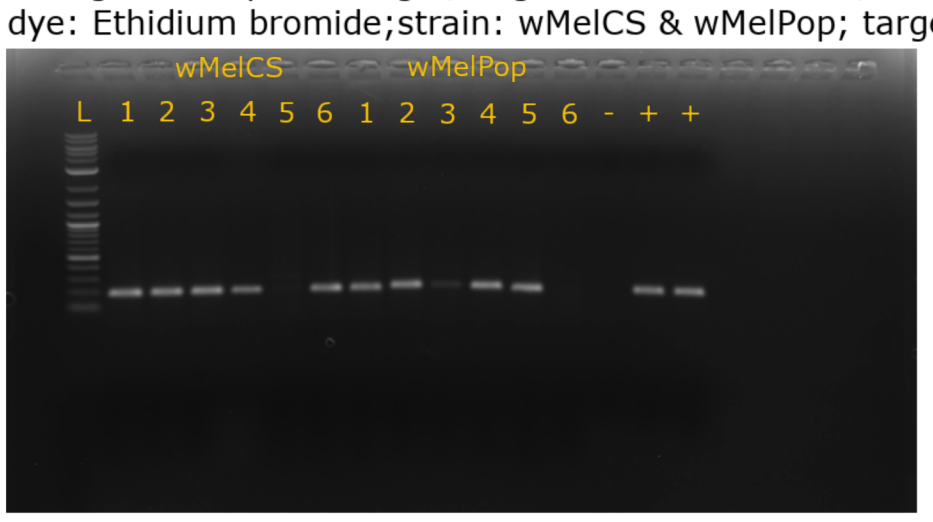

S6 Fig. Electrophoresis gel; migration time: 35 min;
dye: Ethidium bromide;strain: wMelCS & wMelPop; target: inversion mutant

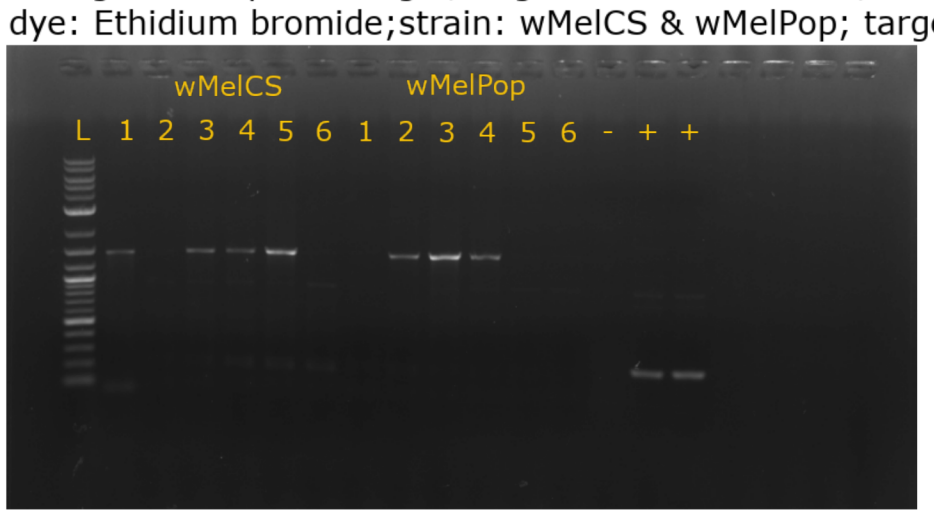

S6 Fig. Electrophoresis gel; migration time: 35 min;
dye: Ethidium bromide;strain: wMelCS & wMelPop; target: inversion reference

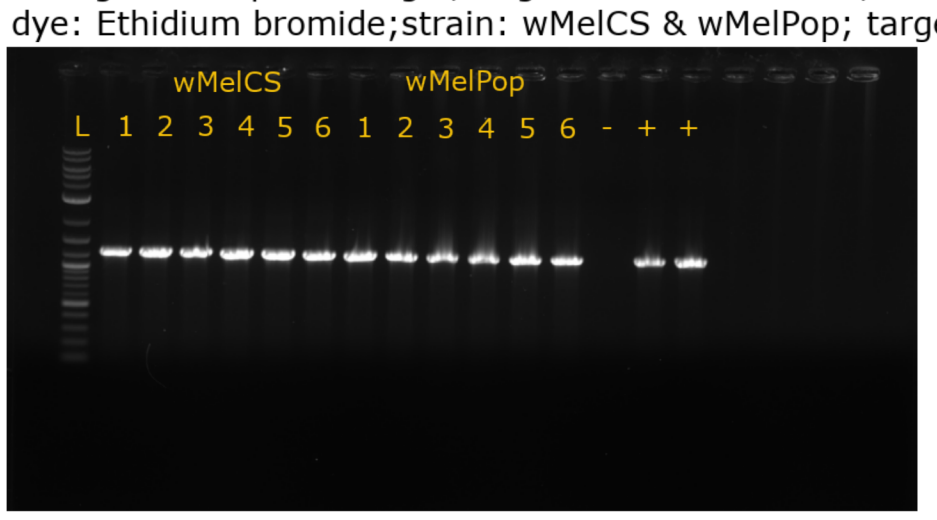

S7 Fig. Electrophoresis gel; migration time: 35 min;
dye: Ethidium bromide;strain: wMelCS & wMelPop; target: inversion mutant

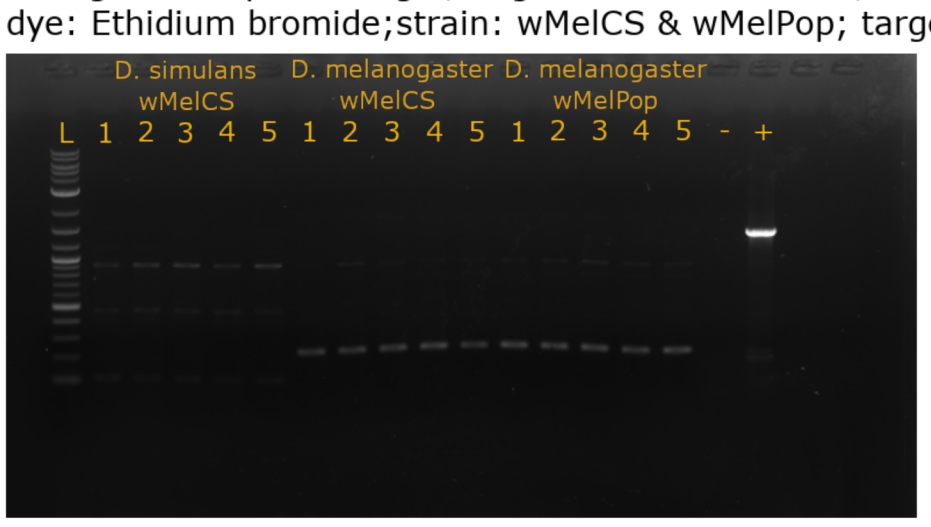

S7 Fig. Electrophoresis gel; migration time: 35 min;
dye: Ethidium bromide;strain: wMelCS & wMelPop; target: inversion reference

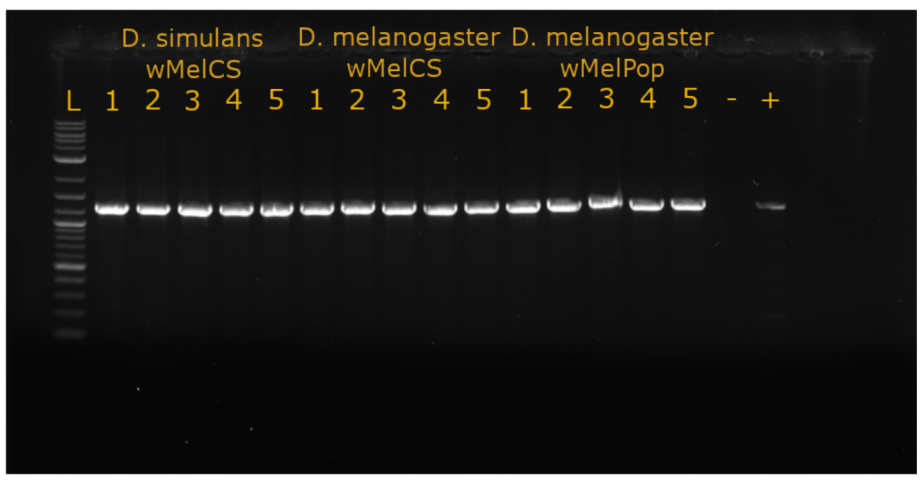

Supplement: Supplementary file 1 [file ppat.1014735.s012.pdf]
