# Supplementary material for: Rapid and convergent evolution of Wolbachia genomes in mosquito cell culture
Source: PLoS Pathog. 2026 Oct 23;22(10):e1014735. doi: 10.1371/journal.ppat.1014735 (PMC13719037; doi:10.1371/journal.ppat.1014735)
Supplement: Supplementary file 2 [file ppat.1014735.s021.pdf]

Dear Editor and Reviewers,

We thank you for the careful and constructive assessment of our manuscript, “Rapid and convergent evolution of *Wolbachia* genomes in mosquito cell culture.” We are grateful for the positive evaluation of the study and for the suggestions that have helped us improve its clarity, interpretation, and positioning within the broader literature.

In response to the reviews, we have substantially revised the manuscript. In particular, we have:

- clarified the experimental design, sequencing strategy, replication and evidence supporting the identified genomic changes;

- moderated causal interpretations of mutation-density associations and consistently described the identified loci as candidate density-associated genes;

- added statistical analyses of temporal changes in symbiont density and their relationship with Octomom copy number;

- expanded the discussion of key limitations, including host-cell evolution, founder effects, qPCR-based density estimates and the absence of longitudinal long-read sequencing;

- clarified our use of the terms convergent, parallel and experimental evolution;

- expanded the discussion of Octomom dynamics, candidate genes, structural variation, effective population size and *Wolbachia* evolution in natural populations;

- revised Figures 2 to clarify the genomic boundaries of structural changes.

- Raw sequencing reads and *Wolbachia* genome assemblies from cell culture are available from NCBI under BioProject PRJNA1019527 (accessions: CP195734.1, CP195735.1, CP195736.1, CP195737.1 and CP195738.1). Data and R code use to perform the statistical analyses of density and genomic changes are available at https://doi.org/10.5061/dryad.w3r228183.

Reviewer comments are reproduced below, followed by our responses in blue font. Changes to the manuscript are indicated by italicized text in this response letter. Line numbers refer to the revised manuscript. Finally, we submitted all original uncropped gel images presented in our study (in figures S5, S6 and S7) as a single supporting information file (S1_raw_images.pdf).

Julien Martinez, on behalf of all authors.

Reviewer 1

Comment 1: Terminology: convergent/parallel evolution and "experimental evolution"

Reviewer: Title: "Rapid and convergent evolution of *Wolbachia* genomes in mosquito cell culture"
I agree with the title, as the manuscript compares *Wolbachia* strains with initially distinct phenotypes that converge toward similar mutations. However, the authors also use the term parallel evolution throughout the manuscript. They should clarify these important concepts. It is also unclear to me whether these experiments should be considered experimental evolution, which differs from evolution under experimental conditions.

Response: We agree that these terms should be clearly defined and used consistently. As highlighted in a recent review on convergent, parallel and repeated evolution (https://pubmed.ncbi.nlm.nih.gov/37724599/), the terms "convergent" and "parallel" evolution have been used inconsistently in the literature. The review proposes a distinction based on ancestral states / phenotypes, with parallel evolution referring to similar outcomes arising from similar ancestral states, whereas convergent evolution refers to similar outcomes arising from distinct ancestral states. The review also proposes "repeated evolution" as a broader term encompassing both types of recurrent evolution.

In the revised manuscript, we have now adopted an analogous distinction at the genomic level. We use "convergent evolution" when similar mutations or genomic changes arised independently in distinct ancestral *Wolbachia* genomes, for example mutations affecting WD0835 across different symbiont strains. We then use "parallel evolution" only when the same genomic change occured independently in replicate cell lines derived from the same ancestral *Wolbachia* strain, as is the case in the second cell-culture experiment (Fig S6). Since in this latter experiment, structural changes (Octomom loss and large chromosomal inversion) occurred both across distinct strains (*w*MelCS and *w*MelPop) and among replicate cell lines within strains (6 replicates per strains) and we also use the term "repeated evolution" when discussing these observations collectively.

We have revised the manuscript accordingly, for example:

lines 199-200: *"Importantly, different Wolbachia genomes independently acquired mutations in the same genes or genomic regions. Such convergent mutations…"*

line 209: *"Repeated TE-mediated structural changes"*

lines 245-247: *"In this experiment, we observed evidence of both convergent evolution, with the same changes occurring independently in different Wolbachia genomes, and parallel evolution, where the same changes occurred in replicate cell lines derived from the same ancestral strain."*

Regarding the use of "experimental evolution", as opposed to "evolution under controlled conditions", we thank the reviewer for raising this important point. We agree that our study differs from an artificial selection experiment in which populations are selected explicitly for a predefined phenotype. However, we believe that "experimental evolution" is still appropriate in its broader and commonly used sense. Kawecki et al. 2012 (https://pubmed.ncbi.nlm.nih.gov/22819306/) define experimental evolution as "the study of evolutionary processes occurring in experimental populations in response to conditions imposed by the experimenter". In our study, we imposed a major environmental and demographic transition by transferring five *Wolbachia* strains from *Drosophila* flies into a common mosquito cell-culture environment, maintained the resulting populations through repeated passages for approximately 3 years, and monitored changes in their genomes and allele frequencies over time. The study was therefore designed explicitly to investigate evolutionary change and evidence of selection in an experimental environment.

Importantly, the corresponding *Wolbachia*-infected fly lines were maintained in parallel throughout the cell culture experiment. Targeted Sanger sequencing at the end of the experiment failed to detect the mutations identified in the cell culture in the donor fly lines, supporting the conclusion that the variants arose or increased in frequency during maintenance in cell culture.

Nevertheless, we recognize that the insect populations were not subjected to the same endpoint whole-genome resequencing and that rare ancestral variants below the detection threshold of Sanger sequencing cannot be excluded. We have clarified this limitation in the revised manuscript. Specifically, we added the following text:

Lines 115-116: *"Here we used an experimental evolution approach to investigate the evolution of several Wolbachia strains following their transfer from Drosophila into a common mosquito cell culture environment."*

Lines 192-195: *"For all other mutations, targeted Sanger sequencing failed to detect them in the D. simulans donor lines which were maintained in parallel throughout the experiment, suggesting that these variants were either acquired or increased in frequency specifically in the cell-culture populations (S4 Fig)"*

Comment 2: correct sentence

Reviewer: "such as how well *Wolbachia* grow inside their host."
This sentence sounds strange to me.

Response: We have revised the sentence for clarity (lines 48-49):

"However, we still know very little about how these bacteria evolve or which genes control important traits, such as their abundance within the host."

Comment 3: rapid evolution of cif genes

Reviewer: In mosquitoes, studies from Weill's group have also demonstrated rapid evolution of cif genes (Namias et al., 2024–2025; Amoros et al., 2026).

Response: We thank the reviewer for drawing our attention to this relevant work. We have expanded the introduction and discussion to acknowledge evidence that particular regions of *Wolbachia* genomes, including prophage-associated cif loci, can evolve rapidly in mosquitoes and natural populations. We have added the suggested references.

The revised text reads (line 103-105):

Lines 101-103: *"However, with the exception of specific genomic regions like Octomom in wMelPop and phage-associated reproductive parasitism genes in Culex mosquitoes that were shown to evolve rapidly [26–28], re-sequencing studies following..."*

Lines 531-534: *"Previous studies have highlighted the important role of TEs in Wolbachia evolution and our experiment largely confirmed this [26,27,60–64]. For instance, TEs within phage-associated genomic islands have been proposed to promote the amplification and diversification of reproductive parasitism genes in Culex mosquitoes [27]"*

Lines 602-605: *"Another key difference is likely to lie in the traits and genomic regions exposed to selection. In our cell lines, we detected no mutation within prophage regions or associated genes involved in reproductive parasitism, despite evidence that these loci can evolve rapidly in insect hosts and natural populations [26,27,68,76,77]"*

Comment 4: misplaced reference

Reviewer: Reference 21 is misplaced.

Response: *We agree. The citation was incorrectly positioned and has now been corrected:*

Lines 108: *"Woolfit et al. [21] observed a rapid burst of genomic changes …*

Comment 5: wTro remains at low density despite a convergent WD0835 mutation

Reviewer: You should comment on the case of wTro, which remains at a very low density while still showing convergent mutations in WD0835.

Response: We agree that *w*Tro provides an important counterexample to a simple causal relationship between variation in WD0835 and increased density. We now emphasize that mutations affecting this gene are evidence of convergent evolution, but do not by themselves establish that WD0835 mutations increase *Wolbachia* density.

We have added the following to the discussion (lines 516-521):

"However, the persistence of wTro at low density compared to other strains, despite the emergence of a mutation in the upstream region of WD0835 demonstrates that changes at this locus are not sufficient, on their own, to produce a high-density phenotype. Their effects may depend on the precise mutation and the symbiont and host genomic backgrounds. Nevertheless, the recurrent targeting of WD0835 suggests that this locus may contribute to adaptation to cell culture independently of any direct effect on symbiont density."

Comment 6: relationship between Octomom copy number and density

Reviewer: "In our cell lines, wMel and wMelCS densities were poorly correlated to fluctuations in Octomom copies, although wMelCS tended to show higher densities with low Octomom copy numbers, as expected."
This sentence seems incorrect. As expected, higher densities are observed in the absence of Octomom amplification, not simply with fewer Octomom copies.

Response: We agree that the original wording was imprecise and confusing. Our intention was to describe periods during which relatively low average Octomom copy number coincided with higher *Wolbachia* density, particularly in *w*MelCS, but this pattern was not consistent across the experiment and was especially weak in *w*Mel.

We have now formally tested the relationship between *Wolbachia* density and Octomom copy number (see new S1 table and S6 FigA), both alone and after accounting for temporal trends. These analyses indicate a non-significant association between the two traits in the first cell culture experiment (Fig 1). In the second experiment using multiple replicate cell lines of *w*MelCS and *w*MelPop maintained in more standardized conditions, with many more datapoints (S6 Figure), we detect a significant association between Octomom copy number and density, however, this could not be fully decoupled from the effect of time on density. We have therefore revised the results to describe these observations more cautiously:

Lines 222-229: *"While we observed no significant association between Octomom copy number and density in this experiment, environmental variation, complex population dynamics in cell culture and evolution of the host cell genome could have affected our ability to detect such a relationship. Moreover, endpoint PCR showed that polymorphism in Octomom copy number persisted for extended periods of time, indicating that our cell lines contained mixtures of genotypes carrying one or more copies of Octomom, or lacking the region entirely (S5 Fig). Finally, changes in density could have been affected by mutations in other genes that were segregating in the wMel and wMelCS-infected cell lines (Fig 1C)."*

Lines 249-255: *"When analysed separately, both time and Octomom copy number were significantly associated with density, with Octomom copies and density being negatively correlated (S6A Fig, S1B Table). However, when both predictors were included in the same model, Octomom copy number was no longer significantly associated with density. Because Octomom copy number was itself strongly correlated with time (wMelCS: $R = -0.65$, $p <$*

0.0001 ; wMelPop: R = -0.73, p < 0.0001), the independent contributions of these two factors to changes in density could not be disentangled."

Comment 7: Figure 2, show the exact sequence and boundaries of the Octomom deletion

Reviewer: You should show the sequence of the evolved Octomom region together with the exact boundaries of the Octomom deletion. The current figure is not sufficiently clear on this point.

Response: We have revised Figure 2 to show the precise sequence context and deletion junction for the evolved *w*Mel and *w*MelCS genomes. Panel 2A and 2B now show the single reverse-transcriptase copy retained after deletion and the new panel 2C shows the alignment of the ancestral and evolved sequences at the deletion boundaries. Since the two reverse-transcriptase copies flanking Octomom are identical in sequence, the precise nucleotide at which recombination occurred cannot be determined from the evolved sequence.

Comment 8: transient amplification and possible mutational bias

Reviewer: I do not understand the proposed link between the decrease in Octomom copy number and the suggested mutational bias.

Response: We agree that the proposed mechanism was not adequately explained. Our reasoning was that the presence of multiple homologous copies of Octomom could provide additional opportunities for recombination, potentially increasing the probability of generating deletion variants. However, our data do not directly demonstrate such a mutational bias.

We have therefore rewritten this passage to make this interpretation explicitly speculative (lines 259-265):

"Interestingly, the drop in Octomom copy number was preceded by a transient increase above one copy in most replicates, suggesting that the presence of multiple homologous copies may provide additional opportunities for recombination, increasing the probability of generating deletion variants. Alternatively, the transient increases and subsequent declines may reflect changes in the relative frequencies of pre-existing copy-number genotypes, however, our data do not allow us to distinguish between these two hypotheses."

Comment 9: mechanism of the chromosomal inversion

Reviewer: Does this demonstrate that the inversion is driven by sequence similarity rather than by transposase activity itself? The distinction between these two mechanisms should be clarified.

Response: We agree with the reviewer that the sequence architecture is more consistent with homologous recombination between repeated elements than with transposase activity itself. We have clarified this distinction and added a new paragraph discussing the different ways in which mobile elements can promote structural evolution (lines 531-542):

"Previous studies have highlighted the important role of TEs in Wolbachia evolution and our experiment largely confirmed this [26,27,60–64]. For instance, TEs within phage-associated genomic islands have been proposed to promote the amplification and diversification of reproductive parasitism genes in Culex mosquitoes [27]. Both the Octomom region and the large chromosomal inversion we identified are flanked by identical TEs and have repeatedly evolved in our cell lines. Comparative and phylogenetic studies suggest that TE-associated evolution is partly attributable to their transpositional activity and horizontal transfer between Wolbachia genomes [62,65–67]. However, a large proportion of Wolbachia TEs are also predicted to be nonfunctional [65], including the two pseudogenized IS5-family elements flanking the chromosomal inversion identified here. Their identical sequences could nevertheless provide substrates for homologous recombination. Thus, in addition to active transposition, recombination between TE sequences may be an important mechanism generating structural variation in Wolbachia genomes."

Comment 10: definition of high- and low-density sister strains

Reviewer: I do not understand how the high- and low-density sister strains were defined. I am not convinced that Tri-2 and Ana exhibit sufficiently marked differences in titers.

Response: We thank the reviewer for pointing out that the basis for this classification was not sufficiently clear. The density differences used to select these strain pairs were measured previously in a controlled Drosophila host genetic background (Martinez et al. 2014). That study detected significant differences in density for each of the three sister-strain comparisons used here, including the Tri-2/Ana pair.

We have now clarified this in the text:

lines 338-339: *"we compared the genomes of closely related Wolbachia strains previously shown to differ in density in Drosophila when measured in a controlled host genetic background."*

lines 369-370: *"We selected three pairs of closely related Wolbachia strains exhibiting significant differences in density in Drosophila, as previously shown in [4]".*

We have also added the statistical groupings from the original density analysis to Fig 4A and revised the figure legend accordingly:

lines 346-348: *"Error bars are standard errors and letters indicate statistically significant differences between strains based on a Tukey's honest significance test on ln-transformed data (modified from [4])."*)

Comment 11: WD0508 mutation and rebound in Octomom copy number

Reviewer: How do you explain that a mutation in an Octomom gene could influence its own amplification rate?

Response: We apologize that the original wording implied that the WD0508 mutation directly altered the amplification rate of Octomom, as this was not our intended interpretation. What we meant is that the mutation occurred within an Octomom-bearing genotype, since WD0508 lies within the region. Its increase in frequency coincided with a transient increase in the population-average Octomom copy number. One possible explanation is that the genotype carrying the WD0508 mutation increased in frequency, which would also increase the average representation of Octomom in the population.

We have now clarified this:

lines 482-488:*"The spread of this mutation coincided with a transient but substantial rebound in average Octomom copy number. Because the mutation occurred within an Octomom-bearing genotype, an increase in the frequency of that genotype would also increase the population-average Octomom copy number. Thus, the temporal association is compatible with selection favouring the WD0508-bearing genotype, although our data do not establish that the WD0508 mutation itself was responsible for its increase in frequency.*

Comment 12: horizontal transfer in isopods

Reviewer: In isopods, multiple injections of purified *Wolbachia* have resulted in systemic infections, demonstrating that *Wolbachia* are capable of horizontal transfer.

Response: We thank the reviewer for highlighting this relevant example. We have added the suggested study to the discussion (lines 581-582) when considering horizontal transmission between cells and the capacity of extracellular *Wolbachia* to establish new infections (Le Clec'h W, Braquart-Varnier C, Raimond M, Ferdy J-B, Bouchon D, et al. (2012) High Virulence of Wolbachia after Host Switching: When Autophagy Hurts. PLoS Pathog 8(8): e1002844.).

Comment 13: embryo homogenization

Reviewer: I would state more clearly that the cells are crushed.

Response: We have revised the Methods accordingly (lines 657-659):

"Embryos were then resuspended in 1 ml of Schneider's media, mechanically crushed and homogenized with a sterile pestle to release intracellular Wolbachia."

Comment 14: "When fully confluent"

Reviewer: I do not understand what is meant here.

Response: We have now clarified this point (lines 660-662):

"Once the mosquito cells formed a confluent monolayer covering the base of the well, mosquito cells were serially passaged..."

Comment 15: contamination by other bacteria

Reviewer: How was contamination by other bacteria assessed? Did this ever occur?

Response: We agree that this quality control procedure should be described. Cultures were routinely inspected microscopically for evidence of bacterial or fungal contamination. In addition, we have now performed a taxonomic analysis of small-subunit ribosomal RNA markers recovered from the PacBio sequencing data showing no detectable contamination.

Lines 667-672:

"Cultures were inspected routinely by microscopy for evidence of bacterial or fungal contamination. In addition, Markadoros (https://github.com/sanger-tol/markadoros) was used to assemble and taxonomically classify small subunit (SSU) ribosomal RNA markers to assess the presence of contaminants in the PacBio sequencing data. Assembled SSU were also compared against the NCBI nt database, and no SSU markers other than Wolbachia 16S and mosquito ribosomal sequences were detected (S7 Table)."

Comment 16: reference supporting the HTH gene

Reviewer: Please provide a reference supporting the choice of the HTH gene.

Response: We have now added the 2015 reference study in which the HTH qPCR primers were designed using a highly conserved regulatory region upstream of the homothorax gene

(lines 683, Molloy JC, Sinkins SP. Wolbachia do not induce reactive oxygen species-dependent immune pathway activation in aedes albopictus. Viruses. 2015;7: 4624–4639).

Comment 17: cells rather than host DNA

Reviewer: These are cells that are removed, not host DNA.

Response: we agree and have corrected the wording in lines 731-733: *"For Illumina sequencing, intact host cells and cellular debris were removed before DNA extraction by passing the supernatant through 5 and 2.7 µm pore size filters."*

Reviewer 2

Major comment 1: Sequencing strategies and timepoints underlying Figure 1B

Reviewer: Although the details of experimental design are cleverly incorporated into Fig. 1A, I am not sure which sequencing strategies/timepoints were used to generate Fig. 1B and all the following ones. Is it comparison of two Illumina experiments or PacBio vs published reference genomes?

Response: We agree that the sequencing design was insufficiently clear. Figure 1B summarizes the full set of mutations identified using PacBio HiFi (end of experiment) and Illumina (intermediate timepoints). Mutation trajectories were subsequently followed across a denser series of samples using allele-specific qPCR or Sanger sequencing.

We have clarified this in Figure 1 legend (lines 144-158):

"(A) Changes in Wolbachia density per cell line following transinfection. The wTri-2-infected cell line was generated five months after the other cell lines. Illumina sequencing was performed after 17 and 27 months in cell culture (12 and 22 months for wTri-2). After 34 months (29 months for wTri-2), all cell lines were frozen for one year and later defrosted for PacBio sequencing. (B) Summary of all mutations detected in each Wolbachia genome by comparing endpoint PacBio HiFi and intermediate-timepoint Illumina reads with the corresponding reference genomes from Drosophila hosts."

Major comment 2: Candidate density regulators rather than demonstrated causal genes

Reviewer: Implication of *Wolbachia* genes in density regulation rests on temporal co-occurrence of a mutation and a density shift. As there is no side-by-side ancestral-vs-derived density comparison in the same host genetic background, the genes identified here can be seen only as candidate density regulators.

Response: We agree with the reviewer. The temporal associations between the spread of mutant alleles and symbiont density does not establish that individual mutations caused the observed density shifts. Therefore, with the exception of Octomom, which role in density regulation is supported by previous experimental studies, the genes identified here should be regarded as candidate density-associated genes requiring functional validation.

We have revised the manuscript to make this distinction clearer. We have also expanded the discussion to acknowledge the potential confounding factors, including evolution of the host genome, variation in cell-population composition, uncontrolled environmental variation and limitations of qPCR-based estimates. We also outline experiments that could directly test the causal effects of the identified mutations:

Lines 435-450: *"Overall, Wolbachia density increased in most of our cell lines during experimental evolution. Although changes in the symbiont genome may have contributed to this pattern, our experimental design does not allow us to establish direct causal relationships between individual mutations and changes in density. Other factors may have influenced symbiont density, including fluctuations in uncontrolled environmental conditions or changes in the relative abundance of infected and uninfected host cells. In addition, because host-cell populations evolved alongside Wolbachia, changes in the host genome may have occurred and interacted with the evolving symbiont. Our qPCR-based estimates also reflect the ratio of bacterial to host DNA rather than a direct measure of bacterial abundance per cell and could be affected by changes in representation of the host target, for example through shifts in host-cell ploidy. Functional validation will therefore be required to determine whether the mutations identified here directly affect Wolbachia density. Such validation could include side-by-side comparisons of ancestral and derived symbiont genotypes in a common, controlled host genetic background, ideally complemented by independent measurements of bacterial abundance at the cellular level. Nevertheless, the recurrent occurrence of mutations at several loci, together with their temporal association with density shifts, identifies a set of candidate genes whose potential roles in symbiont density regulation warrant further investigation."*

Major comment 3: Evolution of host-cell genetics

Reviewer: Host cell genetics was not studied, and cell lines were evolving together with *Wolbachia* strains and likely adapting to the new symbionts. Given fast insect cell genome evolution, this sheds even more doubt on the fact that *Wolbachia* genes regulate host cell density in the system. I would recommend re-phrasing it as host-symbiont associations with higher symbiont densities, not *Wolbachia* mutations driving density changes.

Response: We agree that host genome evolution is an important potential confounding factor, and our experiment cannot attribute changes in symbiont density exclusively to evolution of the bacterial genome. Rather than treating this limitation separately, we have incorporated it into the broader discussion paragraph added in response to the previous comment.

Minor comment 1: Correct wording

Reviewer: I recommend substituting “explored” with “observed”.

Response: we have made this change (Lines 117-118).

Minor comment 2: WD0434 alone versus an interaction with WD0835

Reviewer: why interaction and not WD0434 alone? WD0434 was described as interacting with *Drosophila* proteins before (Hamilton W, Hardy E, López-Madrigal S, Phelps M, Martin M, Newton I. *Wolbachia* uses ankyrin repeats to target specific fly proteins. mBio. 2026 May 13;17(5):e0017226. doi: 10.1128/mbio.00172-26. Epub 2026 Apr 20. PMID: 42003611; PMCID: PMC13170367.). It might be worth discussing it in more detail.

Response: Our interpretation was based on the temporality of density and the spread of mutations in the wTri-2 cell line. We agree that we cannot completely rule out the possibility that WD0434 could have led to higher symbiont density on its own, especially given that the protein was shown to interact with host targets.

Thus, we have revised the result section to better acknowledge this possibility, expanded the discussion on the potential role of WD0434 and cited the relevant study of Hamilton et al. 2026:

Lines 287-294: *“The WD0434 mutation rose to high frequency approximately five months before the sustained density increase, whereas the subsequent spread of the WD0835 mutant allele closely coincided with the phenotypic shift. These temporal patterns are consistent with several hypotheses. Since mutations in WD0835 in the other Wolbachia strains did not produce comparable density changes (Fig 1C), it is possible that it is the interaction between the WD0434 and WD0835 mutations that is responsible for the phenotypic change in wTri-2. Alternatively, WD0434 might be solely responsible for this change but its impact on the population lagged behind the mutation’s spread.”*

Lines 297-299: *“WD0434 is a particularly strong effector candidate since it encodes a protein carrying ankyrin repeat domains shown to interact with multiple host targets [37,38]”*

Lines 497-499: *"Recent work has shown that WD0434 can bind specific host proteins and cause severe toxic phenotypes upon heterologous expression, however its effect on symbiont density is yet to be demonstrated [38]."*

Reviewer 3

Comment 1: Figure 1 replication and error estimates

Reviewer: Fig. 1 is very busy... there are no measures of error displayed. This might be acceptable for clarity in panel 1C, but the legend must clearly explain the replication strategy and the number of experiments performed. In 1A, it should be possible to show standard errors for the density data and/or apply a statistical test to indicate when density changed significantly for each strain.

Response: We agree that the replication structure was not sufficiently clear in Fig 1 legend. Each original strain is represented by a single independently established cell line, and each plotted density estimate is the mean of two technical qPCR replicates. These technical replicates quantify measurement precision but do not constitute biological replication. Consequently, we chose not to display standard errors calculated from the two technical replicates, as doing so could imply a level of biological replication that is not present.

Nevertheless, we agree that a statistical test was needed in order to state whether density changed overtime. Since there is only one biological replicate per timepoint and cell line in Figure 1, testing differences among individual timepoints would be inappropriate. Instead, we tested for an overall temporal trend within each cell line. These analyses showed that in all cell lines, except the *w*Mel-infected one, there was a significant increase in density with time. We have used a similar approach using mixed effect linear models in the second experimental evolution assay conducted on replicate cell lines of *w*MelCS and *w*MelPop in order to take into account the random effect of individual cell lines for a given *Wolbachia* strain.

We have now revised the legend of Figure 1 and added the corresponding analyses to the Results, Methods and new supplementary S1 Table:

Lines 144-158 (Fig 1 legend): *"(A) Changes in Wolbachia density per cell line following transinfection. Lines indicate independent cell lines and each datapoint shows symbiont density in a given sample based on the mean of two technical qPCR replicates…(C) Variation in Wolbachia density (red area; left axis) and estimated frequency of mutant alleles relative to the 16S rRNA control gene (right axis) measured by qPCR and averaged over two technical replicates."*

Lines 132-133: *"Although substantial short-term fluctuations were observed, overall Wolbachia density increased significantly throughout the experiment in all cell lines except the wMel-infected line (S1A Table)"*

Lines 690-700: *"Changes in Wolbachia density over time were analysed separately for each strain using linear regression in R [84]. Since density measurements showed evidence of non-*

normality and heteroscedasticity, Box–Cox analyses were used to identify suitable transformations with the boxcox function in the R package MASS. A square-root transformation (λ ≈ 0.5) was supported across Wolbachia strains and was therefore applied. For the experiment containing several replicate cell lines of the same Wolbachia strain (wMelCS and wMelPop), temporal changes in density were analysed using linear mixed-effects models, with symbiont density as the response variable, timepoint as a continuous fixed effect, and replicate cell line identity as a random effect. The significance of fixed effects was assessed using t-tests using the lmer function as implemented in the R package lmerTest [85]. Where appropriate, the effect of Octomom copy number on density was tested, both alone and in combination with the timepoint effect (S1 Table).”

Comment 2: Octomom copy number and density

Reviewer: Whether the results are “expected” is more of a discussion point and shouldn’t be raised here. It is debatable in any case, since previous studies have linked increasing Octomom copy number with higher symbiont densities. As stated later and shown in Fig. S9, Octomom copy number seems unrelated to density in the current study, which receives relatively little commentary in the Discussion, but accords with prior findings from another lab: see https://pubmed.ncbi.nlm.nih.gov/27661080/. Overall, in the Discussion, the authors could be bolder in asserting that Octomom copy number appears not to be a strong driver of Wolbachia density once a larger range of strains is examined than was originally the case when the first papers on this topic were published.

Response: We agree that our original manuscript did not adequately address the complexity of the relationship between Octomom copy number and *Wolbachia* density, and that the phrase “as expected” was inappropriate in the Results. We have therefore removed this wording, performed additional statistical analyses of the relationship between Octomom copy number and density, expanded the Discussion, and added the suggested reference.

In the first cell culture experiment, which followed single wMel and wMelCS cell lines through time, we detected no significant association between Octomom copy number and Wolbachia density (S1A Table). In the second experiment, involving multiple replicate wMelCS and wMelPop cell lines, Octomom copy number was significantly associated with density when analysed alone (new S1B Table; Fig S6A). However, Octomom copy number also changed strongly with time, and its association with density was no longer significant when time and Octomom copy number were included in the same model. Consequently, the contribution of Octomom loss could not be separated statistically from of other time-dependent processes.

Thus, we agree with the reviewer that our data do not support a simple relationship in which Octomom copy number alone is a strong predictor of *Wolbachia* density across experimental conditions. At the same time, we are cautious about concluding that Octomom is unimportant for density regulation. Previous experimental work has shown that both amplification and complete deletion of Octomom can increase density relative to a single-copy state, meaning that the relationship between population-average Octomom copy number and density may be non-linear. In addition, our cell lines contained mixtures of deleted, single-copy and

amplified Octomom genotypes, and density is likely to depend on genetic and environmental factors beyond Octomom itself.

We have now revised the result and discussion accordingly:

Lines 249-255: *"When analysed separately, both time and Octomom copy number were significantly associated with density, with Octomom copies and density being negatively correlated (S6A Fig, S1B Table). However, when both predictors were included in the same model, Octomom copy number was no longer significantly associated with density. Because Octomom copy number was itself strongly correlated with time (wMelCS: $r = -0.65$, $p < 0.0001$; wMelPop: $r = -0.73$, $p < 0.0001$), the independent contributions of these two factors to changes in density could not be disentangled."*

Lines 453-476: *"Consistent with these findings, the loss of Octomom in our cell lines tended to be associated with increases in Wolbachia density. However, the statistical support for this relationship weakened when time was included in the same models and also varied among experiments. Several factors may explain these results. First, Octomom copy numbers changed strongly over time, making it difficult to distinguish the effect of Octomom loss from that of other time-dependent processes. In experiments where the association was non-significant even without accounting for time, limited replication (single cell line and fewer timepoints, Fig 1) may also have reduced our statistical power. In addition, Octomom-related variation might not be fully captured by population-average copy number alone. Our cell lines comprised mixtures of deleted, single-copy and amplified Octomom genotypes that coexisted for extended periods, and the relationship between mean copy number and density may be non-linear in polymorphic cell lines, as both deleted and amplified variants are expected to reach higher densities than single-copy variants. Moreover, long-read sequencing was performed only at the end of the experiment and we cannot exclude the presence of low-frequency or more complex structural variants within Octomom at intermediate timepoints or in the fly donor populations that may have affected density. Finally, Octomom is unlikely to be the sole determinant of density. Previous work has shown that Wolbachia density regulation also depends on factors such as temperature and symbiont genetic background, independently of Octomom copy number [48]. Therefore, mutations in other density-regulating loci may have contributed to density changes and obscured the specific contribution of Octomom in some cell lines. In addition, the relative synchrony of changes among replicate wMelCS and wMelPop cell lines suggests that some shared environmental factors have influenced Wolbachia density, Octomom copy number or both (S6 Fig). Thus, while our results are consistent with Octomom loss contributing to increased Wolbachia density, they do not allow us to quantify its effect independently of other genetic and environmental changes occurring during prolonged cell culture."*

Comment 3: AT-rich region upstream of WD0835

Reviewer: I would not expect this statement in the results. It is something of a hostage to fortune, as it is a hypothesis that could be easily tested by RT-PCR between strains with differences in this region. Consider this point in the Discussion instead, with suggestions for the how the hypothesis could be tested robustly in future (perhaps by Cappable-Seq – see DOI 10.1099/mgen.0.001542).

Response: We have now moved this speculative interpretation from the results to the discussion. We also described how the role of this putative regulatory region could be investigated and added the suggested reference:

Lines 503-508: *"In addition, the recurrent changes in the AT-rich sequence upstream of WD0835 in our cell lines, together with similar variation among naturally occurring Wolbachia strains, raise the possibility that this sequence is part of a rapidly evolving regulatory region controlling WD0835 expression. Further work comparing WD0835 expression between symbiont strains or mapping transcription start sites using approaches such as Cappable-Seq [55] will be needed to test this hypothesis."*

Comment 4: limited long-read sequencing of Octomom

Reviewer: These variations in Octomom highlight an important limitation of the study that needs addressing on the Discussion. The region can only be observed in its full context using long-read sequencing, which is deployed at the end of the study, but not on the original isolates from flies or at intermediate timepoints. Therefore, potential changes such as individual gene deletions within the region (which could be polymorphic at the population level) wouldn't be detected until the end of the study.

Response: We agree that the absence of longitudinal long-read data limits our ability to resolve the full structural dynamics of Octomom and to exclude low-frequency or complex variants in the donor population or at intermediate timepoints. We have now acknowledged this limitation in the discussion. Nevertheless, our intermediate Illumina data showed relatively uniform coverage changes across the region (wMelCS in Fig S3A), and endpoint long-read sequencing recovered the same whole-region deletion genotype independently in wMel and wMelCS (see new Figure 2C), supporting loss of Octomom as a single unit as the predominant structural change.

Lines 464-467: *"Moreover, long-read sequencing was performed only at the end of the experiment and we cannot exclude the presence of low-frequency or more complex structural variants within Octomom at intermediate timepoints or in the fly donor populations that may have affected density."*

Comment 5: absence of long-read data from the donor inocula and founder effects

Reviewer: The lack of long-read data for the Wolbachia genomes at the time of isolation from the fly stocks is an important limitation of the study. It is assumed that these isolates were identical to the reference genomes; however, the mutations and structural changes identified in the cell culture experiments could have arisen from founder effects, with other polymorphisms present in the inoculum being lost. This must be acknowledged.

Response: We agree that the absence of long-read sequencing directly from the donor fly populations means that low-frequency ancestral variants and founder effects cannot be excluded. We have therefore acknowledged this limitation explicitly in the discussion. However, we also note that the identified variants were undetectable for multiple months (often a year, Fig 1C) after transfer and only increased later during prolonged passage, which is less consistent with an immediate founding bottleneck alone. Moreover, the repeated emergence of similar mutations in independent *Wolbachia* strains and replicate cell lines argues against founder effects being the sole explanation and is more consistent with selection contributing to their spread.

We have added the following text:

Lines 425-432: *"Here, we generated new Wolbachia-infected mosquito cell lines and identified mutations, providing new insights into the evolutionary dynamics of Wolbachia in cell culture. While we cannot exclude the possibility that these variants were present at low frequency in the Drosophila donor populations, the identified mutations were initially undetectable and generally rose in frequency only after prolonged passage in cell culture. Together with the repeated occurrence of similar changes in independent cell lines, this pattern is more consistent with selection contributing to their spread during adaptation to the cell culture environment."*

Comment 6: limitations of qPCR-based density estimates

Reviewer: Some important points are raised here, but they also need to concede the limitation of relying on qPCR for density estimates. The cell line is highly heterogeneous as the authors correctly point out, and the proportions of subpopulations of cells could affect qPCR results in the absence of any calibration from imaging or flow cytometric data. Changes in ploidy in cell lines are very common during subculture and hence representation of the host gene target may vary between subpopulations and/or longitudinally.

Response: We agree that qPCR measures the ratio between bacterial and host target-copy numbers and does not directly measure the number of bacteria per individual cell.

We have now acknowledged these limitations when discussing the potential role of candidate symbiont genes in regulating density (Lines 435-450):

"Overall, Wolbachia density increased in most of our cell lines during experimental evolution. Although changes in the symbiont genome may have contributed to this, our experimental design does not allow us to establish direct causal relationships between individual mutations and changes in density. Other factors not investigated here may have influenced symbiont density, including fluctuations in uncontrolled environmental conditions, changes in the proportion of infected and uninfected host cells within cell lines or evolution of the host genome. It is also possible that our qPCR-based estimates, which reflect the ratio of bacterial to host DNA, may have been affected by changes in the representation of the host target, for example through shifts in host cell ploidy during the experiment. Functional validation will therefore be required to determine whether the mutations identified here directly affect Wolbachia density. Nevertheless, the temporal association of several mutations

with shifts in density, together with recurrent changes affecting the same loci across independently evolving Wolbachia strains, provides a set of candidate genes whose potential roles in symbiont density regulation deserves further investigation."

Comment 7: Broader evolutionary context, the natural wMelCS–wMel inversion, and cif evolution

Reviewer: The evolutionary-rate section needs to be placed in the context of genomic evolution in the wild. The classic studies on the replacement of wMelCS by wMel showed a large inversion flanked by IS elements. The obvious parallel should be highlighted. It is also important to note that cif genes and prophage regions did not mutate in culture, although they can evolve rapidly in the wild.

Response: We agree and have added a section on transposable elements to discuss these parallels. In addition, we now contrast the loci evolving in cell culture with those known to diversify rapidly in nature (see response to next comment).

Lines 530-549: *"Role of mobile elements in structural variation*

Previous studies have highlighted the important role of TEs in Wolbachia evolution and our experiment largely confirmed this [26,27,59–63]. For instance, TEs within phage-associated genomic islands have been proposed to promote the amplification and diversification of reproductive parasitism genes in Culex mosquitoes [27]. Both the Octomom region and the large chromosomal inversion we identified are flanked by identical TEs and have repeatedly evolved in our cell lines. Comparative and phylogenetic studies suggest that TE-associated evolution is partly attributable to their transpositional activity and horizontal transfer between Wolbachia genomes [61,64–66]. However, a large proportion of Wolbachia TEs are also predicted to be nonfunctional [64], including the two pseudogenized IS5-family elements flanking the chromosomal inversion identified here. Their identical sequences could nevertheless provide substrates for homologous recombination. Thus, in addition to active transposition, recombination between TE sequences may be an important mechanism generating structural variation in Wolbachia genomes.

The repeated spread of the chromosomal inversion indicates that it may be advantageous in cell culture, although with the current data we cannot establish whether this impacted symbiont density.

Nevertheless, this is reminiscent of the global replacement of wMelCS by the lower-density strain wMel reported in natural populations of fruit flies [63]. The two strains, while genetically very similar, can be distinguished by a large inversion flanked by IS elements. While this inversion occurs at a different genomic position than the 354-kb inversion identified here, the two cases highlight a common capacity of TEs to mediate large-scale genome restructuring."

Comment 8: Effective population size in cell culture versus insects

Reviewer: differences in effective population sizes between cell culture and whole-insect contexts are postulated to account for the higher mutation rate in cell culture. However, the authors seem to oscillate between a belief that effective population sizes are larger in vitro, or larger in the environment (compare lines 496 and 500-501). The key point is that the effective population size is tiny in insects where transmission is almost exclusively vertical, leading to strong bottlenecks and weak selection exacerbated by Muller's ratchet. In vitro, as the authors have already pointed out, horizontal transmission exists at high frequency in parallel with inheritance of symbiont populations following host cell division; thus, the effective population size is likely to be orders of magnitude greater (for a strain at moderate to high density).

Response: We agree that our previous discussion on differences in evolutionary rates between cell culture and insects may have been ambiguous. We have therefore rewritten this section to clarify our hypotheses for the accelerated rate of detectable genomic change in cell culture, emphasizing the potential importance of transmission mode and recurrent genetic bottlenecks, as suggested by the reviewer.

We have also placed our results in a broader evolutionary context by noting that prophage-associated and reproductive-parasitism genes are known to evolve rapidly in natural *Wolbachia* associations, whereas we detected no mutations in these regions in our cell lines. This highlights a second potential difference between cell culture and insect hosts which is the traits and genomic regions that are under selection.

Lines 587-612: *"All the mutations we characterized in our cell lines were undetectable in the Wolbachia genomes sequenced from their natural Drosophila host species and from the D. simulans lines used for transinfection. Although low-frequency ancestral variants cannot be excluded, this suggests that these Wolbachia strains underwent little genomic change over 6 to 21 years spent in a novel insect host background, yet accumulated multiple mutations over much shorter timescales in cell culture. In line with this, our meta-analysis also showed a consistently higher rate of genomic change in cell culture than in studies conducted in insect hosts.*

These observations suggest that the evolutionary dynamics of Wolbachia, and particularly the relative roles of selection and genetic drift, differ markedly between the two environments. In insects, maternal transmission imposes severe genetic bottlenecks at each host generation, reducing effective population sizes and increasing the influence of genetic drift. In contrast, in cell culture, Wolbachia can be inherited during host cell division while also spreading horizontally between cells. This mixed mode of transmission is expected to lead to higher effective population sizes, thereby reducing the relative influence of drift and increasing the efficacy with which beneficial variants can rise in frequency.

Another key difference is likely to lie in the traits and genomic regions exposed to selection. In our cell lines, we detected no mutation within prophage regions or associated genes involved in reproductive parasitism, despite evidence that these loci can evolve rapidly in

insect hosts and natural populations [26,27,67,75,76]. This may reflect relaxation of selection on reproductive parasitism in cell culture, where host sexual reproduction is absent. Conversely, genes affecting intracellular proliferation of the symbiont may be under strong selection in cell culture, if horizontal transmission of Wolbachia between cells is highly advantageous.

Thus, the contrast between cell culture and insect hosts is unlikely to simply reflect a difference in the overall rate of evolution. Rather, these environments appear to differ both in the efficiency and the targets of selection. Cell culture may therefore lead to evolutionary trajectories that differ qualitatively from those observed in natural host-symbiont associations."

Minor edit 1: Correct sentence

Reviewer: please correct to: "This approach was successful in identifying Octomom, a 21 kb genomic region involved in density regulation in the pathogenic strain wMelPop, which overproliferates at high temperatures in Drosophila melanogaster, causing severe reductions in host longevity [20]."

Response: We have now corrected (Lines 84-86).

Minor edit 2: Reference typo

Reviewer: Please correct to: "Woolfit et al. [21] observed a rapid burst..."

Response: We have now corrected (Lines 108).

Minor edit 3: Adding reference

Reviewer: Please cite https://doi.org/10.1534/g3.115.017137

Response: We have now added the suggested reference in lines 524-527.

Minor edit 4: Superscript formatting for cm^2

Reviewer: Please ensure that numbers are superscripted for cm2.

Response: We have corrected all occurrences of "cm2" to "cm^2" throughout the Methods.
