# Supplementary material for: Rapid and convergent evolution of Wolbachia genomes in mosquito cell culture
Source: PLoS Pathog. 2026 Oct 23;22(10):e1014735. doi: 10.1371/journal.ppat.1014735 (PMC13719037; doi:10.1371/journal.ppat.1014735)
Supplement: Peer review history

# Peer review: Decision Letter 0

**DOI:** 10.1371/journal.ppat.1014735.r001

**Authors:** Debra Bessen, Elizabeth McGraw

20 Jul 2026

PPATHOGENS-D-26-01241

Rapid and convergent evolution of *Wolbachia*  genomes in mosquito cell culture

PLOS Pathogens

Dear Dr. Martinez,

Thank you for submitting your manuscript to PLOS Pathogens. After careful consideration, we feel that it has merit but does not fully meet PLOS Pathogens's publication criteria as it currently stands. Therefore, we invite you to submit a revised version of the manuscript that addresses the points raised during the review process.

Please submit your revised manuscript by Aug 19 2026 11:59PM. If you will need more time than this to complete your revisions, please reply to this message or contact the journal office at plospathogens@plos.org. When you're ready to submit your revision, log on to https://www.editorialmanager.com/ppathogens/ and select the 'Submissions Needing Revision' folder to locate your manuscript file.

Please include the following items when submitting your revised manuscript:

* A letter that responds to each point raised by the editor and reviewer(s). You should upload this letter as a separate file labeled 'Response to Reviewers'. This file does not need to include responses to any formatting updates and technical items listed in the 'Journal Requirements' section below.

* A marked-up copy of your manuscript that highlights changes made to the original version. You should upload this as a separate file labeled 'Revised Manuscript with Track Changes'.

* An unmarked version of your revised paper without tracked changes. You should upload this as a separate file labeled 'Manuscript'.

If you would like to make changes to your financial disclosure, competing interests statement, or data availability statement, please make these updates within the submission form at the time of resubmission. Guidelines for resubmitting your figure files are available below the reviewer comments at the end of this letter.

As the corresponding author, your ORCID iD is verified in the submission system and will appear in the published article. PLOS supports the use of ORCID, and we encourage all coauthors to register for an ORCID iD and use it as well. Please encourage your coauthors to verify their ORCID iD within the submission system before final acceptance, as unverified ORCID iDs will not appear in the published article. Only the individual author can complete the verification step; PLOS staff cannot verify ORCID iDs on behalf of authors.

We look forward to receiving your revised manuscript.

Kind regards,

Elizabeth A McGraw, PhD

Academic Editor

PLOS Pathogens

Debra Bessen

Section Editor

PLOS Pathogens

Sumita Bhaduri-McIntosh

Editor-in-Chief

PLOS Pathogens

orcid.org/0000-0003-2946-9497

Michael Malim

Editor-in-Chief

PLOS Pathogens

orcid.org/0000-0002-7699-2064

**Journal Requirements:**

If the reviewer comments include a recommendation to cite specific previously published works, please review and evaluate these publications to determine whether they are relevant and should be cited. There is no requirement to cite these works unless the editor has indicated otherwise.

1) Please ensure that the CRediT author contributions listed for every co-author are completed accurately and in full.

At this stage, the following Authors/Authors require contributions: Julien Martinez, Anne W Wairimu, and Steven P Sinkins. Please ensure that the full contributions of each author are acknowledged in the "Add/Edit/Remove Authors" section of our submission form.

The list of CRediT author contributions may be found here: https://journals.plos.org/plospathogens/s/authorship#loc-author-contributions

2) We ask that a manuscript source file is provided at Revision. Please upload your manuscript file as a .doc, .docx, .rtf or .tex. If you are providing a .tex file, please upload it under the item type u2018LaTeX Source Fileu2019 and leave your .pdf version as the item type u2018Manuscriptu2019.

3) We do not publish any copyright or trademark symbols that usually accompany proprietary names, eg ©,  ®, or TM  (e.g. next to drug or reagent names). Therefore please remove all instances of trademark/copyright symbols throughout the text, including:

- TM on page: 27.

4) Please upload all main figures as separate Figure files in .tif or .eps format. For more information about how to convert and format your figure files please see our guidelines:

https://journals.plos.org/plospathogens/s/figures

5) Please upload a copy of Figure Fig 1-5 which you refer to in your text on pages 11, 14, 17, 18, and 19. Or, if the figure is no longer to be included as part of the submission please remove all reference to it within the text.

6) Please amend your detailed Financial Disclosure statement. This is published with the article. It must therefore be completed in full sentences and contain the exact wording you wish to be published.

i) State the initials, alongside each funding source, of each author to receive each grant. For example: "This work was supported by the National Institutes of Health (####### to AM; ###### to CJ) and the National Science Foundation (###### to AM)."

ii) State what role the funders took in the study. If the funders had no role in your study, please state: "The funders had no role in study design, data collection and analysis, decision to publish, or preparation of the manuscript."

iii) If any authors received a salary from any of your funders, please state which authors and which funders..

If you did not receive any funding for this study, please simply state: u201cThe authors received no specific funding for this work.u201d

7) PLOS now requires that authors provide the original uncropped and unadjusted images underlying all blot or gel results reported in a submission’s figures or Supporting Information files. This policy and the journal’s other requirements for blot/gel reporting and figure preparation are described in detail at https://journals.plos.org/plosone/s/figures#loc-blot-and-gel-reporting-requirements and https://journals.plos.org/plosone/s/figures#loc-preparing-figures-from-image-files.

When you submit your revised manuscript, please ensure that your figures adhere fully to these guidelines and provide the original underlying images for all blot or gel data reported in your submission. See the following link for instructions on providing the original image data: https://journals.plos.org/plosone/s/figures#loc-original-images-for-blots-and-gels.

In your cover letter, please note whether your blot/gel image data are in Supporting Information or posted at a public data repository, provide the repository URL if relevant, and provide specific details as to which raw blot/gel images, if any, are not available. Email us at customercare@plos.org if you have any questions.

**Reviewers' Comments:**

Reviewer's Responses to Questions

**Part I - Summary**

Please use this section to discuss strengths/weaknesses of study, novelty/significance, general execution and scholarship.

Reviewer #1: The manuscript by Martinez and colleagues is an excellent study addressing the genomic evolution of five *Wolbachia*  strains from supergroup A maintained in the same Aedes albopictus mosquito cell line. The results are extremely robust, both in terms of biological replicates and sequencing approaches, combining Illumina and PacBio sequencing, with Sanger sequencing used to confirm selected mutations. The convergent mutations identified are therefore highly convincing. The broader contextualization of the findings in the meta-analysis presented in Figure 5 is particularly valuable, as it places the results within the general framework of *Wolbachia*  genomic evolution in cell culture, in laboratory insects (during transinfection), and in the field (World Mosquito Program). These findings are entirely novel and represent a major advance in our understanding of the evolutionary mechanisms shaping *Wolbachia*  genomes.

Reviewer #2: The manuscript entitled “Rapid and convergent evolution of Wolbachiagenomes in mosquito cell culture” describes an experiment, where six *Wolbachia*  strains are transinfected from D. simulans into Ae. albopictus Aa23 cells. They track density over ~2.5 years, and re-sequence to catch de novo mutations. They show that (i) *Wolbachia*  evolves fast in cell culture (unlike after host shifts / field release); (ii) Octomom region of *Wolbachia*  genome is repeatedly lost across wMel/wMelCS/wMelPop strains; (iii) new candidate *Wolbachia*  density regulators can be identified in cell culture. The experimental-evolution-as-functional-genetics angle is useful given the absence of *Wolbachia*  transformation tools.

Reviewer #3: This is a very interesting and important study for the *Wolbachia*  field. Methodologically, it is generally well-performed, although the weaknesses of relying on PCR-based methods rather than sequencing for some of the main conclusions require clear acknowledgment. Other key limitations are skirted around in the Discussion, and the placement in the context of prior work is somewhat incomplete.

**********

**Part II – Major Issues: Key Experiments Required for Acceptance**

Please use this section to detail the key new experiments or modifications of existing experiments that should be absolutely required to validate study conclusions.

Generally, there should be no more than 3 such required experiments or major modifications for a "Major Revision" recommendation. If more than 3 experiments are necessary to validate the study conclusions, then you are encouraged to recommend "Reject".

Reviewer #1: (No Response)

Reviewer #2: 1) Although the details of experimental design are cleverly incorporated into fig. 1A, I am not sure which sequencing strategies/timepoints were used to generate fig 1B ad all the following ones. Is it comparison of two Illumina experiments or PacBio vs published reference genomes?

2) Implication of *Wolbachia*  genes in density regulation rests on temporal co-occurrence of a mutation and a density shift. As there is no side-by-side ancestral-vs-derived density comparison in the same host genetic background, the genes identified here can be seen only as candidate density regulators.

3) Host cell genetics was not studied, and cell lines were evolving together with *Wolbachia*  strains and likely adapting to the new symbionts. Given fast insect cell genome evolution, this sheds even more doubt on the fact that *Wolbachia*  genes regulate host cell density in the system. I would recommend re-phrasing it as host-symbiont associations with higher symbiont densities, not *Wolbachia*  mutations driving density changes.

Reviewer #3: In my opinion, the authors don't need to return to the lab as long as they fully acknowledge the study's limitations and put it in the proper context (see below).

**********

**Part III – Minor Issues: Editorial and Data Presentation Modifications**

Please use this section for editorial suggestions as well as relatively minor modifications of existing data that would enhance clarity.

Reviewer #1: Below are a few minor comments, following the chronology of the manuscript:

Title: “Rapid and convergent evolution of *Wolbachia*  genomes in mosquito cell culture”

I agree with the title, as the manuscript compares *Wolbachia*  strains with initially distinct phenotypes that converge toward similar mutations. However, the authors also use the term parallel evolution throughout the manuscript. They should clarify these important concepts.

It is also unclear to me whether these experiments should be considered experimental evolution, which differs from evolution under experimental conditions.

Line 49: “such as how well *Wolbachia*  grow inside their host.”

This sentence sounds strange to me.

Line 105: “In contrast to insect studies, there is emerging evidence that *Wolbachia*  may evolve rapidly when the symbiont is maintained in insect cell culture, potentially due to altered population dynamics and selective pressures in this environment”

In mosquitoes, studies from Weill’s group have also demonstrated rapid evolution of cif genes (Namias et al., 2024–2025; Amoros et al., 2026).

Line 107:

Reference 21 is misplaced.

You should comment on the case of wTro, which remains at a very low density while still showing convergent mutations in WDO835.

Line 212: “In our cell lines, wMel and wMelCS densities were poorly correlated to fluctuations in Octomom copies, although wMelCS tended to show higher densities with low Octomom copy numbers, as expected.”

This sentence seems incorrect. As expected, higher densities are observed in the absence of Octomom amplification, not simply with fewer Octomom copies (fewer Octomom copies = lower densities).

Figure 2: You should show the sequence of the evolved Octomom region together with the exact boundaries of the Octomom deletion. The current figure is not sufficiently clear on this point.

Line 241: “Interestingly, the drop in Octomom copy number was preceded by a transient increase above one copy in most replicates, suggesting a possible mutational bias whereby deletion events are more likely when multiple copies are present”.

I do not understand the proposed link between the decrease in Octomom copy number and the suggested mutational bias.

Line 248: “In addition to Octomom deletion, the large chromosomal inversion identified in wMelCS likely involved TE-mediated recombination, as the region is flanked by two pseudogenized but identical IS5-family transposase-encoding elements”

Does this demonstrate that the inversion is driven by sequence similarity rather than by transposase activity itself? The distinction between these two mechanisms should be clarified.

Line 327:

I do not understand how the high- and low-density sister strains were defined. I am not convinced that Tri-2 and Ana exhibit sufficiently marked differences in titers.

Line 419: “This mutation coincided with a transient but substantial rebound in Octomom copy number, indicating that it may have been adaptive”

How do you explain that a mutation in an Octomom gene could influence its own amplification rate?

Line 420:

In isopods, multiple injections of purified *Wolbachia*  have resulted in systemic infections, demonstrating that *Wolbachia*  are capable of horizontal transfer.

Line 549:

I would state more clearly that the cells are crushed.

Line 551: “When fully confluent”

I do not understand what is meant here.

Line 559:

How was contamination by other bacteria assessed? Did this ever occur?

Line 556:

Please provide a reference supporting the choice of the HTH gene.

Line 604:

These are cells that are removed, not host DNA.

Reviewer #2: Line 116: I recommend substituting “explored” with “observed”.

Lines 272-273 – “Since mutations in WD0835 in the other *Wolbachia*  strains did not produce comparable density changes (Fig 1C), it is possible that it is the interaction between the WD0434 and WD0835 mutations that is responsible for the phenotypic change in wTri-2.” – why interaction and not WD0434 alone? WD0434 was described as interacting with Drosophila proteins before (Hamilton W, Hardy E, López-Madrigal S, Phelps M, Martin M, Newton I. *Wolbachia*  uses ankyrin repeats to target specific fly proteins. mBio. 2026 May 13;17(5):e0017226. doi: 10.1128/mbio.00172-26. Epub 2026 Apr 20. PMID: 42003611; PMCID: PMC13170367.). It might be worth discussing it in more detail.

Reviewer #3: Fig. 1 is very busy, although the display of multiple strands of data across the timeline of the experiments has advantages. On the other hand, there are no measures of error displayed. This might be acceptable for clarity in panel 1C, but the legend must clearly explain the replication strategy and the number of experiments performed. In 1A, it should be possible to show standard errors for the density data and/or apply a statistical test to indicate when density changed significantly for each strain (mark with asterisks at relevant time-points).

Lines 212-214: Whether the results are “expected” is more of a discussion point and shouldn’t be raised here. It is debatable in any case, since previous studies have linked increasing Octomom copy number with higher symbiont densities. As stated later and shown in Fig. S9, Octomom copy number seems unrelated to density in the current study, which receives relatively little commentary in the Discussion, but accords with prior findings from another lab: see https://pubmed.ncbi.nlm.nih.gov/27661080/. Overall, in the Discussion, the authors could be bolder in asserting that Octomom copy number appears not to be a strong driver of *Wolbachia*  density once a larger range of strains is examined than was originally the case when the first papers on this topic were published.

Lines 310-311: It is stated that “Altogether, this suggests that this AT-rich sequence might be a rapidly-evolving promoter region controlling WD0835 expression”. I would not expect this statement in the results. It is something of a hostage to fortune, as it is a hypothesis that could be easily tested by RT-PCR between strains with differences in this region. Consider this point in the Discussion instead, with suggestions for the how the hypothesis could be tested robustly in future (perhaps by Cappable-Seq – see DOI 10.1099/mgen.0.001542).

Lines 331-332: These variations in Octomom highlight an important limitation of the study that needs addressing on the Discussion. The region can only be observed in its full context using long-read sequencing, which is deployed at the end of the study, but not on the original isolates from flies or at intermediate timepoints. Therefore, potential changes such as individual gene deletions within the region (which could be polymorphic at the population level) wouldn’t be detected until the end of the study.

Lines 406-408: The lack of long-read data for the *Wolbachia*  genomes at the time of isolation from the fly stocks is an important limitation of the study. It is assumed that these isolates were identical to the reference genomes; however, the mutations and structural changes identified in the cell culture experiments could have arisen from founder effects, with other polymorphisms present in the inoculum being lost. This must be acknowledged.

Lines 462-471: Some important points are raised here, but they also need to concede the limitation of relying on qPCR for density estimates. The cell line is highly heterogeneous as the authors correctly point out, and the proportions of subpopulations of cells could affect qPCR results in the absence of any calibration from imaging or flow cytometric data. Changes in ploidy in cell lines are very common during subculture and hence representation of the host gene target may vary between subpopulations and/or longitudinally.

The latter part of the discussion gets somewhat confused, especially in the section headed “Evolutionary rate in cell culture versus the insect model”. Although there is a focus on how *Wolbachia*  evolves in new host environments, this can’t be decoupled from what we know about *Wolbachia*  genomic evolution in the wild, much of which is highly relevant to the current study. For instance, the classic studies on the rapid, natural replacement of wMelCS with wMel in D. melanogaster showed that these genomes differ predominantly due to large chromosomal inversion flanked by IS elements (see https://doi.org/10.1016/j.cub.2005.06.069). Presumably, the inversion in wMelCS identified in the current study relates to a different part of the genome (please clarify), but the obvious parallel here should be highlighted. It is also important to note that cif genes (and prophage regions in general) didn’t mutate in culture, while these are parts of the genome expected to undergo the most rapid evolution in the wild (see https://doi.org/10.1093/genetics/iyag097).

In this same paragraph, differences in effective population sizes between cell culture and whole-insect contexts are postulated to account for the higher mutation rate in cell culture. However, the authors seem to oscillate between a belief that effective population sizes are larger in vitro, or larger in the environment (compare lines 496 and 500-501). The key point is that the effective population size is tiny in insects where transmission is almost exclusively vertical, leading to strong bottlenecks and weak selection exacerbated by Muller’s ratchet. In vitro, as the authors have already pointed out, horizontal transmission exists at high frequency in parallel with inheritance of symbiont populations following host cell division; thus, the effective population size is likely to be orders of magnitude greater (for a strain at moderate to high density).

Minor edits

Lines 85-86, please correct to: "This approach was successful in identifying Octomom, a 21 kb genomic region involved in density regulation in the pathogenic strain wMelPop, which overproliferates at high temperatures in Drosophila melanogaster, causing severe reductions in host longevity [20]."

Lines 107-109, please correct to: "Woolfit et al. [21] observed a rapid burst of genomic changes during the passaging of wMelPop in mosquito cell lines, but detected no further mutations after subsequent transinfection into mosquitoes."

Line 445: Please cite https://doi.org/10.1534/g3.115.017137

Paragraph starting line 593: please ensure that numbers are superscripted for cm2.

**********

PLOS authors have the option to publish the peer review history of their article (what does this mean?). If published, this will include your full peer review and any attached files.

If you choose “no”, your identity will remain anonymous but your review may still be made public.

**Do you want your identity to be public for this peer review?**  For information about this choice, including consent withdrawal, please see our Privacy Policy.

Reviewer #1: No

Reviewer #2: No

Reviewer #3: No

[NOTE: If reviewer comments were submitted as an attachment file, they will be attached to this email and accessible via the submission site. Please log into your account, locate the manuscript record, and check for the action link "View Attachments". If this link does not appear, there are no attachment files.]

**Figure resubmission:**

While revising your submission, we strongly recommend that you use PLOS’s NAAS tool (https://ngplosjournals.pagemajik.ai/artanalysis) to test your figure files. NAAS can convert your figure files to the TIFF file type and meet basic requirements (such as print size, resolution), or provide you with a report on issues that do not meet our requirements and that NAAS cannot fix.

After uploading your figures to PLOS’s NAAS tool - https://ngplosjournals.pagemajik.ai/artanalysis, NAAS will process the files provided and display the results in the "Uploaded Files" section of the page as the processing is complete. If the uploaded figures meet our requirements (or NAAS is able to fix the files to meet our requirements), the figure will be marked as "fixed" above. If NAAS is unable to fix the files, a red "failed" label will appear above. When NAAS has confirmed that the figure files meet our requirements, please download the file via the download option, and include these NAAS processed figure files when submitting your revised manuscript.

**Reproducibility:**

To enhance the reproducibility of your results, we recommend that authors of applicable studies deposit laboratory protocols in protocols.io, where a protocol can be assigned its own identifier (DOI) such that it can be cited independently in the future. Additionally, PLOS ONE offers an option to publish peer-reviewed clinical study protocols. Read more information on sharing protocols at https://plos.org/protocols?utm_medium=editorial-email&utm_source=authorletters&utm_campaign=protocols

# Author comment: Author response to Decision Letter 1

**DOI:** 10.1371/journal.ppat.1014735.r002

1 Oct 2026

Attachment Submitted filename: Response to reviewers.pdf

# Editor report: Decision Letter 1

**DOI:** 10.1371/journal.ppat.1014735.r003

**Authors:** Debra Bessen, Elizabeth McGraw

5 Oct 2026

Dear Dr. Martinez,

We are pleased to inform that your manuscript, "Rapid and convergent evolution of *Wolbachia*  genomes in mosquito cell culture", has been editorially accepted for publication at PLOS Pathogens.

Before your manuscript can be formally accepted and sent to production, you will need to complete our formatting changes, which you will receive by email within a week. Please note that your manuscript will not be scheduled for publication until you have made the required changes.

IMPORTANT NOTES

(1) Copyediting and Proofreading: The corresponding author will receive a typeset proof for review, to ensure errors have not been introduced during production. Please review the PDF proof of your manuscript carefully, as this is the last chance to correct any errors. Please note that major changes, or those which affect the scientific understanding of the work, will likely cause delays to the publication date of your manuscript.

(2) Appropriate Figure Files: Please remove all name and figure # text from your figure files. Please also take this time to check that your figures are of high resolution, which will improve the readbility of your figures and help expedite your manuscript's publication. Please note that figures must have been originally created at 300dpi or higher. Do not manually increase the resolution of your files. For instructions on how to properly obtain high quality images, please review our Figure Guidelines, with examples at: http://journals.plos.org/plospathogens/s/figures.

We encourage you to use PACE (http://pace.apexcovantage.com/), a figure-checking tool which helps authors ensure all figures meet PLOS requirements so that figures can publish in the highest quality. PACE is a digital diagnostic and conversion tool for figure files. It will provide information about any failed check(s) and, if able, will automatically convert the figure into an acceptable file that passes quality checks.

To use PACE, you must first register as a user. Then, login and navigate to the UPLOAD tab, where you will find detailed instructions on how to use the tool.

(3) Striking Image: Please upload a striking still image to accompany your article if one is available (you can include a new image or an existing one from within your manuscript). Should your paper be accepted, this image will be considered for our monthly issue image and may also appear on our website to feature your article. Please upload this as a separate file, selecting "striking image" as the file type upon upload. Please also include a separate "Other" file with a caption, including credits and any potential copyright information. Please do not include the caption in the main article file. If your image is from someone other than yourself, please ensure that the artist has read and agreed to the terms and conditions of the Creative Commons Attribution License at http://journals.plos.org/plospathogens/s/content-license. Please note that PLOS cannot publish copyrighted images.

(4) Press Release or Related Media: If you or your institution will be preparing press materials for this manuscript, please inform our press team in advance at plospathogens@plos.org as soon as possible. We ask that you contact us within one week to plan ahead of our fast Production schedule. If you need to know your paper's publication date for related media purposes, you must coordinate with our press team, and your manuscript will remain under a strict press embargo until the publication date and time.

(5) Update your Profile Information: Now that your manuscript has been provisionally accepted, please log into Editorial Manager and update your profile, if needed. Go to http://www.editorialmanager.com/ppathogens, log in, and click on the "Update My Information" link at the top of the page. Please update your user information to ensure an efficient production and billing process.

(6) LaTeX users only: Our staff will ask you to upload a TEX file in addition to the PDF before the paper can be sent to typesetting, so please carefully review our Latex Guidelines http://journals.plos.org/plospathogens/s/latex in the meantime.

Thank you again for supporting Open Access publishing; we are looking forward to publishing your work in PLOS Pathogens.

Best regards,

Elizabeth A McGraw, PhD

Academic Editor

PLOS Pathogens

Debra Bessen

Section Editor

PLOS Pathogens

Dominique Soldati-Favre

Editor-in-Chief

PLOS Pathogens

***********************************************************

# Editor report: Acceptance letter

**DOI:** 10.1371/journal.ppat.1014735.r004

**Authors:** Debra Bessen, Elizabeth McGraw

Dear Dr. Martinez,

We are delighted to inform you that your manuscript, "Rapid and convergent evolution of *Wolbachia*  genomes in mosquito cell culture," has been formally accepted for publication in PLOS Pathogens.

We have now passed your article onto the PLOS Production Department who will complete the rest of the pre-publication process. All authors will receive a confirmation email upon publication.

Thank you again for supporting open-access publishing; we are looking forward to publishing your work in PLOS Pathogens.

Best regards,

Dominique Soldati-Favre

Editor-in-Chief

PLOS Pathogens
